# Effects of medium chain triglycerides supplementation on insulin sensitivity and beta cell function: A feasibility study

**DOI:** 10.1371/journal.pone.0226200

**Published:** 2019-12-23

**Authors:** Dylan D. Thomas, Mary-Catherine Stockman, Liqun Yu, Tova Meshulam, Ashley C. McCarthy, Annaliese Ionson, Nathan Burritt, Jude Deeney, Howard Cabral, Barbara Corkey, Nawfal Istfan, Caroline M. Apovian

**Affiliations:** 1 Department of Medicine, Section of Endocrinology, Diabetes, Nutrition and Weight Management, Boston University, MA, United States of America; 2 Department of Medicine, Section of Endocrinology, Diabetes and Nutrition and Weight Management, Nutrition and Weight Management Center, Boston Medical Center, Boston, MA, United States of America; 3 Boston University School of Medicine, Boston, MA, United States of America; 4 Department of Biostatistics, Boston University School of Public Health, Boston, MA, United States of America; University of Alabama at Birmingham, UNITED STATES

## Abstract

**Objective:**

Medium chain triglycerides (MCT) have unique metabolic properties which may improve insulin sensitivity (Si) and beta cell function but data in humans are limited. We conducted a 6-week clinical trial of MCT oil supplementation.

**Methods:**

22 subjects without diabetes (8 males, 14 females, mean ± standard error age 39±2.9 years, baseline BMI 27.0±1.4 kg/m^2^) were counseled to maintain their body weight and physical activity (PA) during the trial. Dietary intake, PA data, body composition, and resting energy expenditure (REE) were obtained through dietary recall, international PA questionnaire, dual x-ray absorptiometry, and indirect calorimetry, respectively. MCT prescriptions were given based on REE and PA to replace part of dietary fat with 30 grams of MCT per 2000 kcal daily. Insulin-modified frequently sampled intravenous glucose tolerance tests were performed before and after MCT to measure changes in Si, acute insulin response (AIR), disposition index (DI), and glucose effectiveness (Sg).

**Results:**

MCT were well tolerated and weight remained stable (mean change 0.3 kg, p = 0.39). Fasting REE, respiratory quotient, and body composition were stable during the intervention. There were no significant changes in mean fasting glucose, insulin, insulin resistance, fasting total ketones, Si, AIR, DI, Sg, leptin, fructosamine, and proinsulin. The mean change in Si was 0.5 10^−4^ min^-1^ per mU/L (95% CI: -1.4, 2.4), corresponding to a 12% increase from baseline, and the range was -4.7 to 12.9 10^−4^ min^-1^ per mU/L. Mean total adiponectin decreased significantly from 22925 ng/mL at baseline to 17598 ng/mL at final visit (p = 0.02). The baseline clinical and laboratory parameters were not significantly associated with the change in Si.

**Discussion:**

There were a wide range of changes in the minimal model parameters of glucose and insulin metabolism in subjects following 6 weeks of MCT as an isocaloric substitution for part of usual dietary fat intake. Since this was a single-arm non-randomized study without a control group, it cannot be certain whether these changes were due to MCT so further randomized controlled trials are warranted.

## Introduction

Obesity-related prediabetes and type 2 diabetes (T2D), which are associated with insulin resistance and hyperinsulinemia, affected 84.1 million and 30.3 million people in the United States in 2015, respectively [1]. These conditions are associated with substantially increased risk of cardiovascular disease and morbidity, which may be mediated by chronic inflammation. New treatments are needed given the rising prevalence of these conditions around the world [2]. These are complex heterogeneous diseases which have been demonstrated to have different phenotypes and response to therapies in different racial and ethnic groups [3].

Medium chain triglycerides (MCT) have demonstrated some potentially beneficial anti-inflammatory and metabolic properties. Triglycerides are hydrolyzed into free fatty acids (FFA) which amplify beta cell secretion of insulin in response to glucose [4]. Medium chain fatty acids (MCFA) include caproic (C6:0), caprylic (C8:0), capric (C10:0), and lauric acid (C12:0) and contribute less insulinotropic signaling [5] and are associated with reduced inflammation and improved insulin sensitivity [6] compared with long chain fatty acids (LCFA) in vitro.

The longer chain saturated fatty acids palmitate (C16:0) and stearate (C18:0) elevate NFκB signaling and induce insulin resistance in skeletal muscle, but the MCFA caprylate (C8:0) and laurate (C12:0) do not have this effect [6]. MCFA have been associated with enhanced mitochondrial beta-oxidation [7] and biogenesis [8], and increased mitochondrial respiratory capacity and lower oxidative stress [9]. Ketone bodies (acetoacetate and beta-hydroxybutyrate) are elevated following MCT consumption and may act as signaling molecules that inhibit inflammation [10–12].

Epidemiologic studies have shown that dietary consumption of short chain fatty acids and MCFA was associated with a reduced risk of T2D [13]. However, MCT compose only a minor fraction of the dietary intake of most people. In the Malmö Diet and Cancer Cohort, dietary consumption of short chain fatty acids and MCFA with 4–12 carbons (predominantly from dairy) was associated with reduced risk of developing T2D [13]. MCT supplementation as an oil has associated with reduced triglycerides (TG) [14–16] and short term increases in fatty acid oxidation, thermogenesis, and energy expenditure in humans [17–25]. In trials of hypocaloric diets that randomized subjects to MCT or LCT, MCT were associated with greater weight loss [26, 27], fat loss, and improvement in insulin sensitivity [28]. In trials of isocaloric diets, MCT were also associated with a decrease in adiposity [20], inflammation [29], and weight loss [30], although its effects on insulin sensitivity have been mixed [31, 32]. These studies of MCT have varied widely in study populations and protocols and have been limited by short durations. The long term metabolic effects of MCT under isocaloric conditions remain unclear [33]. Studies of MCT in African Americans are warranted given their known metabolic differences and higher risk of diabetes [34].

We performed this study to better characterize the effects of 6 weeks of isocaloric consumption of MCT in subjects without T2D and to define phenotypes of subjects that may benefit from dietary MCT use. In this clinical trial, we assessed the effects of 6 weeks of supplementation with MCT on insulin sensitivity (Si), beta cell function, and adipokines in a population of lean and obese Caucasians and African Americans without diabetes. We also examined subgroups defined by race, sex, and BMI to clarify who might derive greater benefit from MCT.

## Methods

### Study design and subjects

This was an open-label single-arm, non-blinded, non-randomized pilot clinical trial. The study was approved by the Boston University (BU) Medical Campus and Boston Medical Center (BMC) Institutional Review Board. All subjects provided written informed consent. The trial is registered at clinicaltrials.gov with NCT02783703 and is complete. Initial IRB approval was obtained at the BUMC IRB on November 30, 2016. All protocol amendments were approved prior to implementation. The first subject was enrolled (consented) into the study on April 28, 2017 and the last study visit was completed on June 20, 2018. The authors confirm that all ongoing and related trials for this intervention are registered.

Subjects were recruited from the Endocrinology, Diabetes, Nutrition and Weight Management clinic at Boston Medical Center as well as through IRB-approved fliers, online advertisements (BMC and BU nutrition research webpages, Craigslist, ResearchMatch, and BU Study Finder), and all staff and student emails at BMC and BU.

This study consisted of five in-person visits (screening, two baseline visits, and two follow-up visits) with four weekly phone visits between baseline and follow-up.

Participants were ambulatory, English-speaking individuals between the ages of 18 and 65 who self-identified as White/Caucasian or Black/African American with a body mass index (BMI) ≤5.0 kg/m^2^. Although we originally sought to include only subjects with metabolic syndrome by National Cholesterol Education Program Adult Treatment Panel III criteria, recruitment was lower than anticipated and so this was removed from the inclusion criteria in October 2017. Exclusion criteria included T2D or hemoglobin A1c >6.5% at screening; use of insulin, oral hypoglycemic agents, insulin-sensitizing agents, steroids, weight loss medications or sex hormone therapy; unstable weight within 3 months prior to baseline (defined as weight change greater than 3%); use of certain psychotropic medications; chronic kidney disease; poorly controlled cardiovascular disease or congestive heart failure; severe peripheral vascular or liver diseases; current diagnosis of cancer; abnormal TSH level at screening; or any cognitive or other disorders that may have interfered with participation or ability to follow restrictions. Additionally, participants with self-reported severe claustrophobia, weight greater than 450 lb (205 kg) or height greater than 6’6” were excluded due to technical capacities of metabolic equipment used during the study. For safety reasons, participants with untreated anemia or medically required use of anticoagulant therapies were also excluded. The sample size of this first-in-human study was selected to be similar to other human studies of MCT [29]. Subjects were provided with a fair IRB-approved compensation for their time and effort to attend each in-person study visit.

### Screening visit

All subjects provided written informed consent prior to any screening activities. At the screening visit, subjects had their weight, height, waist circumference, and systolic and diastolic blood pressure measured. Fasting weight was measured to the nearest 0.1 kg with a digital scale and height was measured to the nearest 0.1 cm with a wall stadiometer. Waist circumference was measured to the nearest 0.1 cm at the umbilicus, or in case of sagging abdomen at the iliac crest. Medical history and current medications were reviewed. Baseline labs were obtained including basic metabolic panel, complete blood count, TSH, hemoglobin A1c and lipid panel to confirm eligibility if not performed at our institution within 6 months prior to screening. Subjects were instructed to fast overnight (at least 12 hours) and to avoid alcohol and strenuous activity for 24 hours prior to each in-person visit.

### Metabolic assessments

At baseline, a registered dietitian (RD) obtained participants’ dietary intake through 24-hour dietary recall in order to provide subject-specific suggestions for incorporation of MCT oil into their diet throughout the study. The baseline dietary data were semi-quantitative in order to provide a baseline for the dietary modification, and hence were not included in the statistical models. A physical examination was performed. Resting energy expenditure (REE) and body composition were assessed through indirect calorimetry and dual x-ray absorptiometry (DXA), respectively. Physical activity level was determined through the long-form international physical activity questionnaire (IPAQ) [35]. Frequently sampled intravenous glucose tolerance tests (FSIVGTT) were performed to assess Si. Baseline procedures (IPAQ, indirect calorimetry, DXA, and FSIVGTT) were repeated after the MCT oil intervention. Subjects were instructed to fast overnight for at least 12 hours before these visits and to avoid alcohol and strenuous activity for 24 hours before each visit which is the standard protocol for FSIVGTT. Subjects were instructed to avoid aspirin, NSAIDS, anticoagulants, or other compounds (including omega-3 supplements and fish oil) that may affect bleeding, platelets, or bruising for 72 hours.

### Indirect calorimetry

Indirect calorimetry is the gold standard for determining individual caloric needs [36]. The Parvo Medics TrueOne® 2400 Canopy System metabolic cart (Sandy, UT) was utilized for this study. Indirect calorimetry provided REE, respiratory quotient (RQ), volume of oxygen (VO2), and volume of carbon dioxide (VCO2) which are measured every 30 seconds. Total energy expenditure (TEE) was calculated by multiplying the REE obtained through indirect calorimetry by the physical activity factor determined through the IPAQ. Calorimtery was performed for 15 minutes and data from the final 10 minutes were used to assess the RQ, VO2, and VCO2.

### Body composition assessment

Whole body fat mass, fat-free mass, and android and gynoid fat were assessed with DXA using the GE Healthcare Lunar iDXA™ (Chicago, IL).

### Dietary intervention

MCT oil [shorter than C8:0 < 1%, C8 (Octanoic acid) 54%, C10 (Decanoic acid) 41%, longer than C10 < 5%, Nestle, Switzerland] was given based on TEE to replace part of dietary fat with 30 grams of MCT per 2000 kcal daily (249 kcal/2000 kcal or 12.5% of calculated TEE). This dose is similar those used in other human studies of MCT that showed significant physiological effects on energy expenditure and weight [22, 26, 34].

Following the baseline FSIVGTT, subjects were given the MCT oil and instructed to use it in drinks, salads and/or for light meal preparation needs over the 6-week study period. Bottles were weighed prior to dispensing and weights recorded on an accountability log. Individual dietary modifications were provided to maintain usual caloric intake with the goal of minimally altering the baseline macronutrient composition of the participants’ diets. Subjects were counseled to maintain their body weight and physical activity level throughout the study. For the following four weeks, subjects had weekly phone calls with the study dietitian. The RD estimated and reinforced compliance, provided dietary adjustments as needed, delivered weight maintenance counseling, and monitored for adverse events. At week five, subjects returned for another assessment with indirect calorimetry, DXA, and IPAQ. At week six, subjects returned for their final FSIVGTT, when they returned the MCT oil bottles. Returned bottles were weighed, and compliance was calculated as the ratio of measured oil consumed over expected oil consumed.

### FSIVGTT

Insulin-modified FSIVGTT were performed before and after MCT use. A glucose bolus dose of 300 mg/kg body weight was administered intravenously and blood was sampled for glucose and insulin concentrations at -20, -15, -10, -5, 1, 2, 3, 4, 5, 6, 8, 10, 12, 14, 16, 19, 22, 25, 30, 40, 50, 60, 70, 80, 90, 100, 110, 120, 140, 160, and 180 minutes relative to the bolus. In order to improve the accuracy of the test, insulin (Humulin® R) was administered at a dose of 0.025 unit/kg body weight at the 20 minute mark [37]. Body weight used for the doses was obtained from the prior in-person visit, approximately seven days before. A fasting blood sample was drawn prior to each FSIVGTT for serum lipids, fructosamine, leptin, proinsulin, and total adiponectin. Serum was stored at -80°C until it was analyzed.

### Lab assays

Serum glucose was measured with YSI STATPlus^TM^ 2900 (Yellowsprings, OH). Insulin was assayed with the STELLUX Chemiluminescent Human Insulin ELISA (Alpco, Salem, NH). Proinsulin was assayed with the STELLUX Chemiluminescent Human Total Proinsulin ELISA (Alpco, Salem, NH).

Fructosamine, which is a measure of glycemia of 2–3 weeks duration [38], was assessed with the Fructosamine Colorimetric Kit (Biovision, Milpitas, CA). Leptin was measured with the Invitrogen eBioscience Human Leptin Instand ELISA Kit (Fisher, Pittsburgh, PA). Total adiponectin was measured with the Adiponectin Human ELISA Kit (Fisher, Pittsburgh, PA). Fasting total cholesterol, high-density lipoprotein-cholesterol (HDL), low density lipoprotein-cholesterol (LDL), and triglycerides (TG) were measured in our institution’s clinical laboratory using the Abbott Architect c8000 (Abbott Park, IL). Beta-hydroxybutyrate and acetoacetate were measured as previously reported [39, 40].

Bergman Minimal Model was run with the MinMod Millenium software (Los Angeles, CA) to estimate parameters from the FSIVGTT glucose and insulin data [41]. The main parameters are: Si index; glucose effectiveness (Sg) as a measure of the ability of glucose to enhance its own clearance; and the acute insulin response (AIR) as a measure of beta cell function. The disposition index (DI) represents insulin secretion adjusted for Si (DI = AIR x Si). The beta cell demand index (BCDI) is ratio of AIR / Si and represents quantitative measure of the position of AIR on the hyperbola relative to the Si scale with higher values representing a greater burden of beta cell secretion. In addition, insulin resistance was calculated with the Homeostatic Model Assessment of Insulin Resistance (HOMA-IR) [42].

The predefined primary outcomes were the changes in Si, Sg, AIR, and DI from baseline to final visit. Predefined secondary outcomes included changes in body weight, blood lipids, resting energy expenditure, body composition, blood pressure, and waist circumference.

### Statistical analyses

Data were analyzed with an intention to treat strategy regardless of compliance. As an exploratory analysis, we defined a meaningful increase in Si as a ≥ 10% increase such as might occur following a modest lifestyle intervention [43], and this change could have clinically significant effects over time [44, 45]. A meaningful decrease in Si was defined as a ≥ 10% decrease in Si. Mean changes pre- and post-MCT were assessed with paired two-tailed t-tests and between exploratory subgroups were assessed with two-tailed t-tests. A multivariate regression was performed that assess the factors that were associated with the change in Si. All analyses were performed in SAS Studio 3.8 (Cary, NC).

## Results

**Fig 1:**
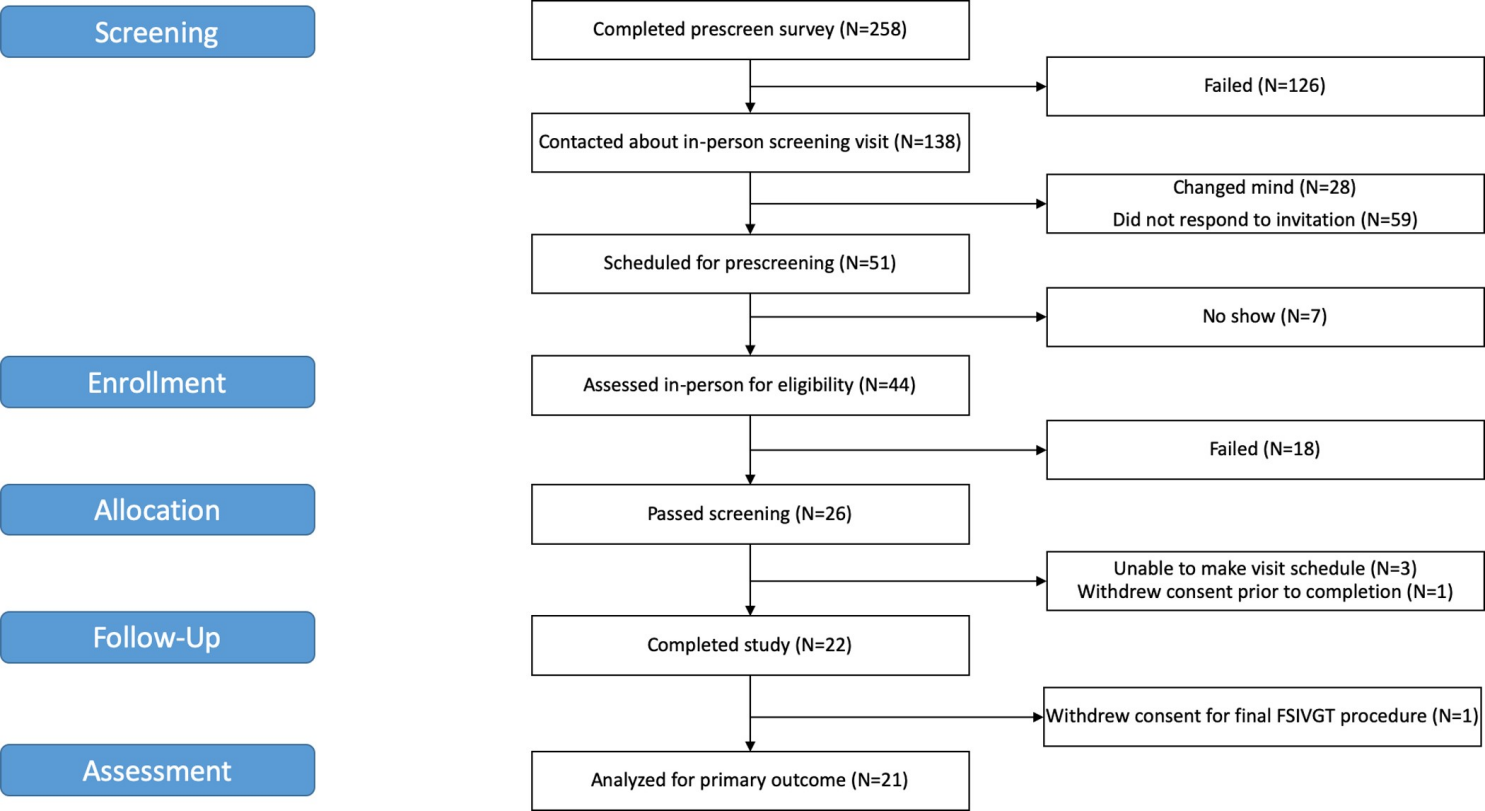
CONSORT flow diagram of medium chain triglyceride study participants.

Forty-four subjects provided informed consent and were screened for eligibility, and 26 subjects were determined eligible to participate (Fig 1). As a result of participant scheduling conflicts, 3 subjects withdrew from the study and one withdrew consent. Twenty-two subjects (14 females, 8 males) completed the baseline FSIVGTT (Table 1). Of these 22 subjects, 16 self-identified as non-Hispanic Caucasian, and six identified as non-Hispanic African American. At baseline mean weight was 78.5±4.6 kg (range: 44.6–145.6 kg) and mean hemoglobin A1C was 5.2±0.1% (range: 4.6–6.1%). There were no major deviations from the study protocol. Recruitment and follow-up visits were conducted between April 27, 2017 and June 20, 2018.

**Table 1:** Baseline demographic and metabolic parameters of study participants (n = 22).

**Age (years)**		38.7 (2.8)
**Sex, n (%)**	Female	14 (63.6)
	Male	8 (36.4)
**Self-identified ethnicity, n (%)**	Non-Hispanic	21 (95.5)
	Hispanic	1 (4.6)
**Self-identified race, n (%)**	Black	6 (27.3)
	Caucasian	16 (72.7)
**Weight (kg)**		78.5 (4.6)
**BMI (kg/m^2^)**		26.9 (1.4)
**Hemoglobin A1C (%)**		5.2 (0.1)
**Daily MCT dose (grams)**		40.7 (2.0)

Data are mean (standard error).

Mean MCT dose was 40.7±2.0 grams or 21% of total daily kilocalories on average. Compliance with the prescribed MCT dose assessed at the final visit was 89±4%. Several subjects reported gastrointestinal distress during the first week of MCT use, but distress subsided with further guidance from the RD to incorporate MCT oil into regular meals and to avoid drinking MCT oil by itself and no patients withdrew from the trial due to these symptoms.

Weight remained stable overall during the trial with a mean weight change of 0.27 kg (95% CI: -0.36, 0.90) with a range of -2.6 to 2.9 kg. Mean fasting insulin, fasting glucose, HOMA-IR, fasting ketones, indirect calorimetry parameters, body composition, blood lipids, leptin, and proinsulin remained stable during the trial (Table 2). Systolic blood pressure, diastolic blood pressure, and mean waist circumference remained stable. The mean change in total adiponectin was -22958 (95% CI: -9893, -986) ng/mL. There was an increase in the mean resting heart rate from the baseline FSIVGTT visit of 68.0±1.1 beats per minute to the final FSIVGTT visit of 71.6±1.4 beats per minute corresponding to a mean change of 3.7 beats per minute (95% CI: 0.75, 6.6).

**Table 2:** Changes in calorimetry, dual x-ray absorptiometry, lipids, labs, and minimal model parameters following 6 weeks of medium chain triglyceride oil supplementation (n = 21).

		Baseline	Final	P-value
**Weight**		78.5 (4.6)	78.8 (4.6)	0.50
**BMI**		26.9 (1.3)	27.0 (1.3)	0.48
**Waist circumference (cm)**		87.5 (4.0)	87.9 (3.6)	0.78
**IPAQ**		1.5 (0.02)	1.5 (0.03)	0.33
**Blood pressure (mm Hg)**		115.5 (2.1)	119.2 (2.0)	0.16
**Resting heart rate**		67.3 (1.2)	71.9 (1.4)	0.02
**Calorimetry**	**VO2 (ml/min)**	256 (11.1)	260 (13.0)	0.64
	**VCO2 (ml/min)**	190 (8.0)	200 (10.9)	0.224
	**RQ**	0.74 (0.01)	0.77 (0.01)	0.232
	**REE (kcal/day)**	1740 (75)	1778 (90)	0.512
**DXA**	**Android (%)**	34.3 (3.4)	34.3 (3.4)	0.611
	**Gynoid (%)**	35.7 (2.3)	35.7 (2.3)	0.646
	**Total fat (%)**	32.9 (2.4)	32.9 (2.4)	0.395
	**Fat free mass (kg)**	49.2 (2.4)	49.6 (2.4)	0.12
**Lipids**	**Total cholesterol (mg/dL)**	196.2 (7.0)	191.5 (7.3)	0.29
	**LDL-C (mg/dL)**	121.7 (7.6)	116.5 (8.1)	0.25
	**HDL-C (mg/dL)**	58.5 (3.6)	58.8 (3.3)	0.81
	**Triglycerides (mg/dL)**	80.9 (8.7)	81.5 (7.7)	0.92
**Fasting labs**	**Fasting insulin (μIU/mL)**	5.0 (0.8)	5.6 (1.2)	0.42
	**Fasting glucose (mg/dL)**	91.1 (3.2)	88.5 (2.9)	0.42
	**HOMA-IR**	1.2 (0.2)	1.2 (0.2)	0.77
	**Total ketones (micromole/L)**	319.0 (61.4)	215.8 (59.5)	0.22
	**Total adiponectin (ng/mL)**	22925 (3011)	17598 (2355)	0.02
	**Fructosamine (μmol/L)**	181.4 (5.1)	187.1 (7.2)	0.5
	**Leptin (ng/mL)**	10.0 (3.3)	10.5 (3.0)	0.83
	**Proinsulin (pmol/L)**	5.02 (0.91)	5.49 (1.24)	0.54
**Minimal model parameters**	**Si (10**^**−4**^ **min**^**-1**^ **per mU/L)**	4.26 (0.60)	4.78 (0.91)	0.57
	AIR (mU/L*min)	612.7 (123.7)	547.9 (124.1)	0.47
	**DI**	2010.9 (388.4)	1862.4 (371.8)	0.61
	**Sg (min**^**-1**^**)**	0.023 (0.002)	0.025 (0.003)	0.27

Data are mean (standard error)

### Overall FSIVGTT results

Our subjects showed a wide heterogeneity in the change in minimal model parameters from baseline to the final visit (Table 2). The mean change in Si was 0.5 10^−4^ min^-1^ per mU/L (95% CI: -1.4, 2.4), corresponding to a 12% increase from baseline, and the range was -4.7 to 12.9 10^−4^ min^-1^ per mU/L. The mean change in disposition index was -148 (95% CI: -746, 449) and the range was -3893 to 2426. The mean change in AIR was -65 mU/L*min (95% CI: -250, 121) and the range was -1329 to 425 mU/L*min. The mean change in Sg was 0.003 min^-1^ (95% CI: -0.002, 0.007) and the range was -0.014 to 0.029 min^-1^.

### Subgroup FSIVGTT results

In African Americans, the mean change in Si was 2.1 10^−4^ min^-1^ per mU/L (95% CI: -1.8, 6.1) and the range was -1.6 to 9.1 10^−4^ min^-1^ per mU/L. In Caucasians, the mean change in Si was -0.1 10^−4^ min^-1^ per mU/L (95% CI: -2.4, 2.2) and the range was -4.7 to 12.9 10^−4^ min^-1^ per mU/L. In men, the mean change in Si was 1.9 10^−4^ min^-1^ per mU/L (95% CI: -3.9, 7.8) and the range was -2.8 to 12.9 10^−4^ min^-1^ per mU/L. In women, the mean change in Si was -0.2 10^−4^ min^-1^ per mU/L (95% CI: -1.6, 1.2) and the range was -4.7 to 3.7 10^−4^ min^-1^ per mU/L. In subjects below the median BMI of 24,6, the mean change in Si was 1.8 10^−4^ min^-1^ per mU/L (95% CI: -2.2, 5.8) and the range was -4.1 to 12.9 10^−4^ min^-1^ per mU/L. In subjects above the median BMI of 24.6, the mean change in Si was -0.6 10^−4^ min^-1^ per mU/L (95% CI: -1.9, 0.6) and the range was -4.7 to 2.5 10^−4^ min^-1^ per mU/L. These subgroups analyses had limited power and due to small sample sizes, and so these differences were not statistically significant (S1 Table).

9 subjects had a ≥ 10% increase in Si, and 10 subjects had a ≥ 10% decrease in Si with MCT. Subjects who had a ≥ 10% increase in Si had a significantly higher mean total ketones at baseline compared with subjects who had a ≥ 10% decrease in Si (506.7 micromole/L vs. 140.6 micromole/L, p = 0.002), as well as significantly lower mean fasting glucose (84.5 mg/dL vs. 98.2, p = 0.042, S2 Table).

In a multivariate regression, there were no factors that were significantly associated with the change in Si (Table 3).

**Table 3:** Multivariate regression of change in minimal model insulin sensitivity.

		Parameter estimate (standard error)	P-value
**Age (years)**		-0.03 (0.07)	0.62
**Sex**	Female	Reference	
	Male	3.8 (2.3)	0.12
**Fasting plasma glucose (mg/dL)**		-0.14 (0.19)	0.47
**BMI (kg/m^2^)**		-0.13 (0.16)	0.44
**Hemoglobin A1C (%)**		4.4 (3.7)	0.25
**Baseline fasting total ketones (micromole/L)**		0.006 (0.004)	0.15

## Discussion

To our knowledge, this study represents the first assessment in humans of the effects of 6 weeks of isocaloric MCT use at 12.5% of TEE on insulin sensitivity and beta cell function assessed by FSIVGTT and plasma total adiponectin levels. In our study of a heterogeneous group of ambulatory subjects with and without obesity and metabolic dysfunction, some subjects showed an increase in total adiponectin and insulin sensitivity, and others showed the opposite. Indeed, we observed changes in individual subjects’ Si over 6 weeks ranging from large increases to large decreases despite subjects maintaining a stable weight and body composition. Previously, Eckel and colleagues studied a relatively homogenous population with obesity and T2D and found that MCT improved insulin sensitivity in patients who already have metabolic dysfunction in a monitored inpatient setting [31]. Because our patients varied considerably in their baseline clinical and laboratory parameters, we conducted subgroup analyses to determine whether race, sex, or BMI could distinguish the subjects who had an improvement in Si from the subjects who had a decrease in Si.

Subjects who had a ≥ 10% increase in Si had lower fasting glucose and higher fasting ketones compared with subjects who had a ≥ 10% decrease in Si. These subjects who had higher fasting ketones at baseline may have had a higher rate of ketogenesis or a lower rate of ketolysis due to different baseline dietary patterns such as a lower carbohydrate intake (although our dietary recalls do not suggest this) or other unknown factors. Given that this is an exploratory analysis, we cannot draw causal conclusions from this. Furthermore, in a multivariate analysis there was no significant association between baseline fasting ketones and the change in Si.

Earlier studies of MCT were limited by small sample size [32], short duration [29, 31], or use of a hypocaloric protocol which obscured the effect of MCT due to concurring weight loss [26, 28, 30, 46]. St-Onge et al. studied a higher dose of MCT than used here (30% of energy intake) in an isocaloric crossover study in overweight men and women for 28 days and found an increase in energy expenditure in both sexes but a decrease in adiposity only in men [20] and no change in body composition in women [18]. Our study population was predominantly female (63.6%) and our mean BMI of 26.9 was lower which may account for some of the lack of changes in body composition that we observed.

The net effects of MCT on inflammation and adiponectin isoforms are not clear. A study that randomized subjects with T2D to 14 days of MCT (28% of estimated TEE) or LCT in an isocaloric diet found a reduction in ceramides, sphingomyelin, and acylcarnitines (which have been implicated as mediators of insulin resistance and in the development of diabetic cardiomyopathy) and improved systolic function in subjects with T2D [29]. We did not assess the lipidomic profile in our study. The significant drop in mean total adiponectin that we observed is concerning given adiponectin’s beneficial metabolic and anti-inflammatory properties [57]. However, some of our subjects had an increase in total adiponectin which was associated with an increase in insulin sensitivity during our intervention. Studies that compared long-term MCT and LCT feeding in rats showed that MCT were associated with increased adiponectin along with a reduced adiposity [58, 59]. However, there is also some in vitro evidence that MCT can downregulate adiponectin mRNA expression in adipocytes in the presence of TNF-alpha [60]. Supplementation with n-3 long chain polyunsaturated fatty acids has been associated with an increase in adiponectin [61], and supplementation with MCT has been associated with a decrease in eicosapentaenoic acid (EPA) and docosapentaenoic acid (DHA) [62]. We did not measure changes in EPA and DHA, although this may represent a potential mechanism for the decrease in total adiponectin that we observed.

MCT have complex biological effects that may be tissue specific and vary over time. MCT are directly absorbed into the portal circulation and do not require chylomicron formation hence they are rapidly metabolized into ketone bodies. Long chain triglycerides (LCT), which are the dominant type of dietary fat, are more slowly absorbed into chylomicrons. MCFA can be detected in the circulation in the 1–2 hours following ingestion of 0.5 g per kg of MCT in healthy controls [47]. Acute MCT feeding has been associated with a significant rise in RQ compared with LCT for 6 hours in lean subjects [48]. This may be mediated by a rise in insulin and a drop in plasma glucose which has been seen following MCT feeding in rats and humans [25, 49]. Insulin promotes lipid storage, inhibits fat oxidation, which is associated with an increase in RQ. In healthy subjects, consumption of 48 g of MCT oil after an overnight fast was associated with a greater increase in VO2 consistent with a higher postprandial thermogenesis and a greater increase in insulin compared with consumption of corn oil [25]. In contrast, in a crossover trial of overweight men, 20 g MCT during a standardized liquid meal was not associated with greater insulin secretion than 20 g of corn oil [50]. In a study of overweight men, breakfast administration of 20 g of MCT compared with LCT was associated with decreased caloric intake at lunch, diminished postprandial rise in TG and glucose, and increased peptide YY and leptin [50]. Unlike in that trial, we assessed lipids and leptin in only the fasting state at least 12 hours after the last dose of MCT, and it remained stable of the course of our study which was expected given that the mean weight and body composition remained stable in our subjects. In rats, a high fat, MCT-enriched diet was associated with reduced muscle TG in rats, whereas a high fat, LCT-enriched diet was associated with increased liver TG [51]. We are not able to distinguish between muscle and hepatic insulin sensitivity in our data. Hence, in the short term, MCT have complex dose- and time-dependent effects on gut, adipose, hepatic, and pancreatic tissues.

The long term effects of low dose MCT are less understood, although there is evidence for gradual adaptive changes in their absorption with prolonged use [21, 52]. High dose MCT ingestion is associated with a transient rise in the circulating ketones for several hours [53] even if it given together with dextrose and in subjects with T2D [54] and enhanced postprandial thermogenesis. However, these transient increases in postprandial energy expenditure and basal metabolic rate are seen after 7 days of MCT use, but diminished after 14 days of MCT use [21]. Consistent with this, we did not see changes in fasting RQ or REE over 6 weeks. We did find a slight decrease in fasting ketones which may be due to upregulation of ketolytic enzymes although these were not quantified in this study. The duration of MCT supplementation is important given that adaptive changes have been seen with prolonged ketosis including enhanced fatty acid utilization, ketolysis, and altered IL-6 secretion [55]. A progressive decrease in ketones with dietary MCT was seen for the entire duration of a 44-day study in rats; this may be due to adaptive changes to the MCT-enriched diet, such as reduced hepatic ketone body production or increased peripheral utilization [56]. In a trial that provided 24% of energy intake (800 kcal diet) as MCT (23 g / day) for 4 or 12 weeks, there was also no significant change in ketones [28]. Hence, this adaptive process may not be complete after 6 weeks, and studies of longer duration may show different responses to MCT than we observed here.

Beta-hydroxybutyrate has been associated with different effects on sympathetic nervous system activity in different animal models [63, 64]. The 3.6 beat per minute increase in the resting heart rate (p = 0.0163) that we observed from the baseline to the final visit is of uncertain clinical significance, and given the lack of a control group we cannot conclude that this is purely due to an effect of MCT.

Our trial has several limitations. We did not have a control group so we cannot be certain that the changes observed are purely attributable to MCT instead of randomness. Compliance with the prescribed MCT dose could not be directly verified but was estimated during weekly phone visits and measured by weighing the bottles at the final visit. On average, subjects consumed 89% of the prescribed dose. The use of standard medicine cups to measure MCT oil with intervals of 5 mL was restrictive in that they may have prevented participants from consuming a precise dose. The MCT oil bottles did not have pour spouts, so some oil may have been lost to spillage while measuring. This could have accounted for variations in compliance with the prescribed dose of MCT. The 6-week duration of our trial is longer than most trials of MCT but not as long as some that showed a beneficial effect of MCT on waist circumference [65] and body composition [26]. Our protocol used a lower dose of MCT than other studies but well within the range of prior studies that had shown short-term effects [22, 26, 29, 34]. Our study lacked a run-in period during which participants could have followed a standardized diet. We only measured total adiponectin and did not assess adiponectin isoforms. Lastly, our participants were predominantly young, female, and without metabolic syndrome or T2D which may account for some of the lack of benefits that we observed and limit the generalizability of our findings. Strengths of our study include frequent RD contact which was helpful to ensure that subjects maintained a stable weight, which reduced the potentially confounding effect of weight changes on our outcomes.

## Conclusions

In this pilot single-arm study, six weeks of isocaloric substitution of usual dietary fat with MCT oil at 12.5% of energy needs in weight stable Caucasian and African American adults without T2D was generally well-tolerated and feasible. Although there were no statistically significant changes in overall mean insulin sensitivity over the course of the trial, we observed changes in insulin sensitivity in subgroups including men, African Americans, and subjects with a BMI under 24.6 kg/m^2^ that are hypothesis generating and warrant further study.

## Supporting information

S1 Table Change in minimal model parameters following 6 weeks of medium chain triglyceride supplementation stratified by sex, race, and BMI. Stratification by BMI was not a pre-specified analysis in the protocol.
(DOCX)

S2 Table Clinical and laboratory differences between subjects who had a ≥ 10% increase in Si and subjects who had a ≥ 10% decrease in Si following 6 weeks of medium chain triglyceride supplementation. This is an exploratory that was not pre-specified in the protocol.
(DOCX)

S1 Study dataset (XLSX)

S1 Protocol (PDF)

S1 Trend statement checklist (PDF)
