# Supplementary material for: Effects of medium chain triglycerides supplementation on insulin sensitivity and beta cell function: A feasibility study
Source: PLoS One. 2019 Dec 23;14(12):e0226200. doi: 10.1371/journal.pone.0226200 (PMC6927614; doi:10.1371/journal.pone.0226200)
Supplement: S1 Table — Stratification by BMI was not a pre-specified analysis in the protocol. (DOCX) [file pone.0226200.s001.docx]

**S1 Table. Change in minimal model parameters following 6 weeks of medium chain triglyceride supplementation stratified by sex, race, and BMI. Stratification by BMI was not a pre-specified analysis in the protocol.**

| Sex | Men (n=7) | | | Women (n=14) | | |
| --- | --- | --- | --- | --- | --- | --- |
|  | Baseline | Final | Change | Baseline | Final | Change |
| Si (10^-4^ min^-1^ per mU/L) | 5.24  (1.21) | 7.18  (2.47) | 1.94  (2.38) | 3.78  (0.67) | 3.59  (0.40) | -0.19  (0.66) |
| AIR (mU/L*min) | 541.1 (168.7) | 484.3 (165.1) | -56.8  (185.2) | 648.5 (168.7) | 579.6 (170.3) | -68.9  (101.7) |
| DI | 2746  (945) | 2129  (885) | -617  (604) | 1643  (333) | 1729  (366) | 86  (305) |
| Sg (min^-1^) | 0.0293 (0.0044) | 0.0323 (0.0069) | 0.0030  (0.0032) | 0.0194 (0.0021) | 0.0219 (0.0029) | 0.0025  (0.0032) |
| Race | **Caucasian (n=15)** | | | **African American (n=6)** | | |
|  | Baseline | Final | Change | Baseline | Final | Change |
| Si (10^-4^ min^-1^ per mU/L) | 4.62  (0.79) | 4.50  (1.04) | -0.12 (1.08) | 3.36  (0.68) | 5.48  (1.95) | 2.12  (1.54) |
| AIR (mU/L*min) | 578.0 (162.4) | 571.1 (169.8) | -7.0  (105) | 699.3 (165.9) | 489.9 (113.2) | -209.5  (167.6) |
| DI | 1935  (510) | 1735  (477) | -200  (239) | 2202  (530) | 2182  (562) | -19  (864) |
| Sg (min^-1^) | 0.0239 (0.0025) | 0.0249 (0.0038) | 0.0009  (0.0025) | 0.0196 (0.0047) | 0.0265 (0.0055) | 0.007  (0.005) |
| BMI | **Below median**  **BMI 24.6 (n=10)** | | | **Equal to or greater than median BMI 24.6 (n=11)** | | |
|  | Baseline | Final | Change | Baseline | Final | Change |
| Si (10^-4^ min^-1^ per mU/L) | 4.35  (0.75) | 6.16  (1.66) | 1.80  (1.75) | 4.18  (0.96) | 3.54  (0.75) | -0.64  (0.55) |
| AIR (mU/L*min) | 531.7 (141.8) | 395.2 (136.0) | -136.5  (144.8) | 686.3 (202.1) | 686.6 (199.0) | 0.3  (110.2) |
| DI | 1981  (409) | 1831  (664) | 82  (327) | 1238  (276) | 1994  (479) | -358  (466) |
| Sg (min^-1^) | 0.0243 (0.0027) | 0.0280 (0.0039) | 0.0037  (0.0032) | 0.0212 (0.0035) | 0.0229 (0.0047) | 0.0017  (0.0034) |

Data are mean (standard error)
