# Supplementary material for: Effects of medium chain triglycerides supplementation on insulin sensitivity and beta cell function: A feasibility study
Source: PLoS One. 2019 Dec 23;14(12):e0226200. doi: 10.1371/journal.pone.0226200 (PMC6927614; doi:10.1371/journal.pone.0226200)
Supplement: S2 Table — This is an exploratory that was not pre-specified in the protocol. (DOCX) [file pone.0226200.s002.docx]

**S2 Table. Clinical and laboratory differences between subjects who had a ≥ 10% increase in Si and subjects who had a ≥ 10% decrease in Si following 6 weeks of medium chain triglyceride supplementation. This is an exploratory that was not pre-specified in the protocol.**

|  | | | Change in Si | |  |
| --- | --- | --- | --- | --- | --- |
|  | | | ≥ 10% decrease | ≥ 10% increase | P-value |
| N | | | 10 | 9 |  |
| Sex, n(%) | Male | 5 (50.0) | | 2 (22.22) | 0.21 |
|  | Female | 5 (50.0) | | 7 (77.78) |  |
| Self-identified race, n(%) | Black | 1 (9.09) | | 3 (33.33) | 0.21 |
|  | Caucasian | 10 (90.91) | | 6 (66.67) |  |
| Age (years) | | | 42.3 (4.0) | 36.8 (4.7) | 0.38 |
| Baseline weight (kg) | | | 83.8 (8.7) | 69.3 (4.7) | 0.17 |
| Baseline BMI (kg/m^2^) | | | 27.4 (2.1) | 24.52 (1.5) | 0.29 |
| Baseline hemoglobin A1C (%) | | | 5.27 (0.09) | 5.12 (0.14) | 0.39 |
| Baseline cholesterol (mg/dL) | | | 204.8 (9.6) | 181.8 (11.7) | 0.14 |
| Baseline triglycerides (mg/dL) | | | 90.9 (13.0) | 72.3 (15.0) | 0.36 |
| Baseline LDL-C (mg/dL) | | | 132.2 (11.1) | 106.1 (11.2) | 0.12 |
| Baseline HDL-C (mg/dL) | | | 54.5 (5.0) | 61.3 (5.9) | 0.39 |
| Baseline Waist circumference (cm) | | | 92.5 (7.1) | 80.4 (5.3) | 0.20 |
| Baseline RQ | | | 0.73 (0.02) | 0.76 (0.03) | 0.35 |
| Baseline REE (kcal / day) | | | 1800 (123) | 1642 (101) | 0.34 |
| Baseline total body fat (%) | | | 35.3 (2.8) | 29.4 (3.9) | 0.23 |
| Daily MCT dose (g) | | | 43.0 (3.1) | 38.4 (2.7) | 0.29 |
| MCT compliance (%) | | | 91.5 (5.0) | 88.4 (4.6) | 0.66 |
| Change in total adiponectin (ng/mL) | | | -7830 (1915) | -1511.5 (4251) | 0.20 |
| Baseline total ketones (micromole/L) | | | 140.6 (21.0) | 506.7 (105.2) | 0.002 |
| Final total ketones (micromole/L) | | | 255.8 (121.4) | 181.2 (40.0) | 0.57 |
| Baseline Si (10^-4^ min^-1^ per mU/L) | | | 5.46 (1.02) | 3.12 (0.59) | 0.070 |
| Baseline AIR (mU/L*min) | | | 563.4 (119.6) | 710.9 (262.1) | 0.62 |
| Baseline DI | | | 2806 (699) | 1238 (276) | 0.060 |
| Baseline Sg (min^-1^) | | | 0.024 (0.003) | 0.0220 (0.004) | 0.73 |
| Baseline fasting insulin (μIU/mL) | | | 6.3 (1.2) | 4.1 (1.3) | 0.22 |
| Baseline fasting glucose (mg/dL) | | | 98.2 (4.6) | 84.5 (4.1) | 0.042 |

Data are mean (standard error)
