# Supplementary material for: Effects of medium chain triglycerides supplementation on insulin sensitivity and beta cell function: A feasibility study
Source: PLoS One. 2019 Dec 23;14(12):e0226200. doi: 10.1371/journal.pone.0226200 (PMC6927614; doi:10.1371/journal.pone.0226200)
Supplement: S1 Protocol — (PDF) [file pone.0226200.s004.pdf]

BOSTON UNIVERSITY MEDICAL CENTER

CLINICAL STUDY PROTOCOL

**Study Title:**	*Evaluation of medium chain triglycerides in a mixed racial population of patients: a feasibility study*
**Sponsor:**	Boston Medical Center Department of Medicine<br>Boston University Clinical & Translational Science Institute
**Protocol ID:**	H-35267
**IND No:**	N/A
**Medical Monitor:**	N/A
**Protocol Version / Date:**	Version 12.0 April 2, 2018

CONFIDENTIALITY STATEMENT

The information contained in this document, particularly unpublished data, is the property of Boston University Medical Center, and is provided to you in confidence as an investigator, potential investigator, or consultant, for review by you, your staff, and an applicable Institutional Review Board or Independent Ethics Committee.

The information is only to be used by you in connection with authorized clinical trial described in the protocol. You will not disclose any of the information to others without written authorization from Boston University Medical Center, except to the extent necessary to obtain informed consent from those persons to whom the intervention may be administered.

INVESTIGATOR STATEMENT

I have read the protocol, including all appendices, and product information. I agree that they contain all necessary details for me and my staff to conduct this study as described. I will conduct this study as outlined herein and will make a reasonable effort to complete the study within the time designated.

I will provide all appropriate study personnel under my supervision copies of the protocol and access to all information provided by Boston University Medical Center. I will discuss this material with them to ensure that they are fully informed about the drugs and the study.

[signature] 4/9/2018

Signature *Date*

Caroline Apovian MD

Printed Name

TABLE OF CONTENTS

PROTOCOL SYNOPSIS 6

1 STUDY OBJECTIVES 7

2 INTRODUCTION 8

2.1 BACKGROUND 8

2.1.1 MCT influence glucose metabolism 8

2.1.2 Animal studies of MCT show numerous metabolic benefits 8

2.2 HUMAN STUDIES OF MCT SHOW THEY ARE SAFE AND EFFECTIVE 8

2.2.1 MCT may beneficially affect glucose metabolism 8

2.2.2 MCT are safe 8

2.2.3 MCT have an effect on obesity and MS 9

2.2.4 Racial disparities highlight the potential benefit of MCT in AA 9

3 STUDY DESIGN 11

4 OUTCOME MEASURES 12

4.1 PRIMARY OUTCOME 12

4.1.1 Insulin Sensitivity and Bergman's Minimal Model analyses. 12

4.1.2 Disposition Index (DI) and Beta Cell Demand Index (BCDI). 12

4.2 SECONDARY OUTCOMES 12

4.2.1 Weight Change 12

4.2.2 Body Composition 12

4.3 SAFETY MEASURES 12

4.3.1 Pregnancy 12

5 SUBJECT SELECTION 13

5.1 NUMBER OF PARTICIPANTS 13

5.2 INCLUSION CRITERIA 13

5.3 EXCLUSION CRITERIA 13

5.4 RESTRICTIONS DURING THE COURSE OF THE STUDY 13

5.5 PARTICIPANT RECRUITMENT AND SCREENING 14

6 STUDY PRODUCT 15

6.1 PRODUCT STORAGE 15

6.2 BLINDING 15

7 STUDY VISITS AND PROCEDURES 16

7.1 SCREENING 16

7.1.1 Pre-Screening 16

7.1.2 Visit 1: Screening 16

7.2 VISITS 2 AND 3: BASELINE 16

7.3 VISITS 4-7 17

7.4 VISITS 8 AND 9: END-OF-INTERVENTION 17

7.5 Study-specific Procedures 18
7.5.1 Medical history *18*
7.5.2 Concomitant medications *18*
7.5.3 Anthropometrics *18*
7.5.3.1 Weight 18
7.5.3.2 Height 18
7.5.3.3 Waist Circumference 18
7.5.4 Vitals *19*
7.5.5 Physical exam *19*
7.5.5 Laboratory tests *19*
7.5.6 Indirect calorimetry *19*
7.5.7 Physical Activity *19*
7.5.8 DXA Scan *19*
7.5.9 Urine Pregnancy Test *20*
7.5.10 Frequently Sampled Intravenous Glucose Tolerance Test *20*
7.5.11 Dietary Counseling *20*
7.5.12 Dispensing Study Product *21*
7.5.13 Adverse Event Monitoring *21*
7.5.14 Product Accountability *21*
7.5.15 Subject Payments *22*
7.5.16 Medical Record Review *22*

8 STATISTICAL PLAN 23

8.1 Data Analysis 23
8.2 Sample Size Justification 23

9 SAFETY MONITORING AND ADVERSE EVENTS 24

9.1 Definitions 24
9.1.1 Adverse Event *24*
9.1.2 Serious Adverse Event *24*
9.1.3 Unanticipated Problem *24*
9.2 Adverse Event Reporting Period 24
9.3 Preexisting Condition 24
9.4 Post-study Adverse Event 25
9.5 Abnormal Laboratory Values 25
9.6 Hospitalization, Prolonged Hospitalization or Surgery 25
9.7 Pregnancy 25
9.8 Recording of Adverse Events 25
9.9 Potential Risks to Participants 26
9.9.1 Blood Draws *26*
9.9.2 MCT Oil *26*
9.9.3 DXA Scans *26*
9.9.4 Questionnaire *26*
9.9.5 FSIVGTT *27*
9.10 Procedures for Protecting Against Potential Risks 27
9.10.1 Blood draws *27*

9.10.2 MCT Oil ... 27
9.10.3 DXA ... 27
9.10.4 FSIVGTT ... 27
9.10.5 Therapeutic Misconception ... 27
9.10.6 Breach of Confidentiality ... 27
9.11 Data and Safety Monitoring Plan ... 28

10 DATA MANAGEMENT ... 29

10.1 General Guidelines ... 29
10.2 Discontinuation of Participants ... 29

11 ADMINISTRATIVE PROCEDURES ... 30

11.1 Compliance with the Protocol and Protocol Revisions ... 30
11.2 Informed Consent ... 30
11.3 Informed Consent Procedures ... 30
11.4 Participants Unable to Give Informed Consent ... 30
11.5 Records and Reports ... 30
11.6 Institutional Review Board (IRB) ... 31
11.7 Record Retention ... 31

APPENDIX 1. SCHEDULE OF EVENTS ... 32

APPENDIX 2. INTERNATIONAL PHYSICAL ACTIVITY QUESTIONNAIRE ... 33

REFERENCES ... 39

PROTOCOL SYNOPSIS

**Title of Study:**	*Evaluation of medium chain triglycerides in a mixed racial population of patients: a feasibility study*
**Study Principal Investigator:**	Caroline M. Apovian, MD
**Associate Investigators:**	Nawfal Istfan, MD, PhD<br>Dylan Thomas, MD<br>Chi Tang, MD<br>Gitanjali Srivastava, MD
**Study Center:**	Single site – Boston University Medical Center
**Total Number of Participants:**	Approximately 24 men and women
**Study Design:**	Open-label feasibility, non-randomized, without placebo-control
**Objectives:**	To determine whether medium chain triglycerides affect insulin secretion dynamics and insulin sensitivity in an isocaloric, six-week intervention study
**Co-Primary Outcomes:**	• Insulin secretion and disposition index assessed by Frequently sampled intravenous glucose tolerance test (FSIVGTT)<br>• Feasibility of the study design to provide data to address the study objective
**Secondary Outcomes:**	• Insulin sensitivity<br>• Beta cell function<br>• Body composition measured by DXA
**Duration of Intervention:**	6 weeks
**Study Product, Dose, Route, Regimen:**	Nestlé (Glendale, CA) Medium chain triglyceride oil 30 gram / 2000 kcal / day by mouth daily

1 STUDY OBJECTIVES

It is generally accepted that type 2 diabetes (T2D) arises from the progression of insulin resistance (IR), with hyperinsulinemia (HI) as a compensatory response. The possibility that HI can precede and contribute to insulin resistance (IR) and metabolic syndrome (MS) has been suggested but not tested in humans. While IR and HI are closely associated, demonstrating a primary role for HI in T2D is key to the development of new treatment strategies for this disease. One group in which HI could play a bigger role in T2D is African Americans (AA) who are known to be more hyperinsulinemic than Caucasian Americans (CA). Racial disparities in T2D treatment outcomes adversely affect AA. Our main hypothesis is that suppression of HI will contribute to the prevention and treatment of T2D, especially among AA. Our goal in this pilot study is to show that consumption of medium chain triglycerides (MCT) in the diet will decrease basal insulin secretion and HI, and will lead to improvement in the insulin sensitivity index (Si). Our specific aims in the current pilot study are:

Aim 1. Determine the effects of MCT on insulin secretion and clearance, insulin sensitivity, beta cell function (BCF), and the disposition index (DI).

24 subjects (12 AA, 12 CA) will participate in a clinical trial in which they will receive MCT for 6 weeks. Insulin secretion dynamics and insulin sensitivity will be assessed by use of the frequently sampled intravenous glucose tolerance test (FSIVGTT) and Bergman's minimal model analysis.

Aim 2. Determine racial differences in the metabolic and clinical responses to MCT between AA and CA subjects

Data from these two aims will be utilized to design a larger clinical trial with the goal of improving T2D treatment by dietary and pharmacologic therapies that directly target HI.

Aim 3. Test the feasibility of the study design to provide data to address Aims 1 and 2

2 INTRODUCTION

This document is a protocol for a human research study to be conducted according to US and international standards of Good Clinical Practice (FDA Title 21 part 312 and International Conference on Harmonization guidelines), applicable government regulations and institutional research policies and procedures.

2.1 Background

2.1.1 MCT influence glucose metabolism

About 62-65% of the fatty acids in coconut oil are MCT. MCT are comprised of caproic (C6:0), caprylic (C8:0), and capric acid (C10:0), and lauric acid (C12:0) and have shorter carbon chains than long chain triglycerides (LCT) that contain 13-21 carbons. MCT are hydrolyzed into medium chain fatty acids (MCFA); LCT are hydrolyzed into long chain fatty acids (LCFA). MCFA also appear to activate peroxisome proliferator-activated receptors (PPAR) and to enhance mitochondrial beta-oxidation[1]. Octanoate (MCT) compared to oleate (LCT) is stored less by adipocytes and oxidized more. Caprylic acid interacts with ghrelin to form acetylated ghrelin, which is involved with growth hormone regulation, glucose homeostasis and adiposity.

2.1.2 Animal studies of MCT show numerous metabolic benefits

MCT feeding of rats led to increased serum and adipose tissue adiponectin. In diabetic ob/ob mice, MCT have been shown to decrease hyperglycemia compared to LCT. Along these lines, MCT decreased insulin resistance and inflammation in diet induced obesity (DIO) mice. MCT have been shown to increase basal lipolysis and decrease stimulated lipolysis in rat adipocytes. Compared to LCFA, administration of MCFA to rats led to reduced skeletal muscle triglycerides and increased oxidative capacity[2].

2.2 Human studies of MCT show they are safe and effective

2.2.1 MCT may beneficially affect glucose metabolism

IR has been associated with defective intramyocellular lipid metabolism and decreased oxidative phosphorylation[3]. These defects may be partially corrected by MCT, which have been shown to decrease insulin resistance[4,5]. A study of 16 obese women who were randomly assigned to a MCT- or LCT-based 800 calorie diet for 4-12 weeks showed improved insulin sensitivity measured by the hyperinsulinemic-euglycemic clamp in the MCT group[6]. In a randomized trial of 10 patients with non-insulin dependent diabetes who were fed an isocaloric diet, MCT reduced post-prandial hyperglycemic excursions by 45% and improved insulin mediated glucose disposal as assessed by euglycemic clamp, compared with LCT[7]. However, in an ambulatory setting of five patients with non-insulin dependent diabetes, a 30 day diet of MCT vs. LCT, the MCT diet produced decreased postprandial glucose excursions but no significant differences in serum fructosamine, fasting insulin, hepatic glucose output, or insulin-mediated glucose metabolism assessed by 6 hour euglycemic clamp[8]. This study was limited by its small sample size and only included subjects with diabetes, unlike our proposal which will focus on subjects who do not have diabetes.

2.2.2 MCT are safe

Lauric acid (C12:0) has been shown in a meta-analysis to significantly decrease the total cholesterol:HDL ratio when it replaces carbohydrates as a source of calories. A study of 28 non-diabetic insulin-resistant men randomized to 20 grams/day of MCT or corn oil found no significant differences between these groups in plasma lipids, TRL apo B-48- and apo B-100-containing lipoprotein kinetics, or the intestinal expression of genes involved in lipoprotein and fatty acid

metabolism. In a hypocaloric weight loss diet trial subjects were randomly assigned to MCT or olive oil, total cholesterol, fasting glucose, and diastolic blood pressure improved and showed no significant differences between the two groups[9]. Plasma ceramides, sphingomyelin, and acylcarnitines have been implicated in the development of diabetic cardiomyopathy and have been shown to decrease in subjects with T2D with MCT supplementation[10].

2.2.3 MCT have an effect on obesity and MS

Overweight women who were treated with MCT vs. LCT for 27 days showed increased fat oxidation and energy expenditure with MCT. Indirect calorimetry of 7 healthy subjects after consuming 48g of MCT oil or corn oil after an overnight fast showed a greater increase in oxygen consumption with MCT consistent with a higher metabolic rate[11]. MCT have been shown to increase energy expenditure and decrease adiposity in a crossover randomized trial of 24 overweight men[12]. MCT increased fat oxidation compared to LCT, which was associated with a small amount of weight loss[13].

A study of free-living obese Chinese subjects with T2D that used 18 g of MCT compared with LCT daily for 90 days found that MCT induced weight loss, decreased waist circumference, and improved insulin sensitivity[5]. MCT can decrease visceral fat mass in non-obese subjects[14]. In a randomized controlled trial of 54 overweight patients with DM, a MCT fat spread was shown to significantly reduce waist circumference by 1.8 cm over 12 weeks[15]. In overweight men, breakfast administration of 20 g of MCT was compared with LCT and found to decrease caloric intake at lunch, decrease the rise in triglycerides and glucose following a meal, and increase peptide YY and leptin[16].

2.2.4 Racial disparities highlight the potential benefit of MCT in AA

In the United States, AA have increased T2D compared to CA, worse glycemic control, and more diabetes-associated complications including retinopathy and nephropathy. The state of "upregulated" BCF and HI in AA may contribute to these racial disparities in T2D[17]. Basal insulin secretion is significantly increased in AA with T2D to degrees not accounted for by IR[18]. After an oral glucose challenge, ethnic differences are seen in insulin sensitivity and insulin response with increased fasting insulin in AA as well as increased insulin response to glucose in AA compared to CA[19]. AA have also been shown to have higher fasting glucagon-like peptide 1 (GLP-1) and GLP-1 response to an oral glucose tolerance test than CA even at comparable levels of insulin sensitivity, fat mass, and leptin[20]. Hence there may also be racial differences in incretin kinetics, entero-insular and entero-hepatic axis regulation, and hepatic insulin clearance.

This is the first study that differentiates the effects of MCT on basal insulin secretion and glucose-stimulated secretion in AA and CA. This will provide scientific support for the novel concept that HI and defective oxidative phosphorylation is a primary etiologic factor in the pathogenesis of MS, IR and T2D. This may lead to a paradigm shift away from the current dogma that HI is a compensation for IR. This pilot project will provide support for a larger clinical trial designed to test a novel therapeutic approach in a racially mixed patient population at risk for T2D. This work is important because of the epidemic increase in obesity and subsequent T2D worldwide[21]. This study will provide a basis for Endocrinology fellows at BUMC to engage in clinical research specifically targeting nutritional medicine approaches to improve insulin resistance and prevent T2D and gather pilot data to submit for future grant applications.

We hope to prove that MCT supplementation is metabolically beneficial and improves the disposition index. IR and HI are central to the development and progression of MS and can be ameliorated with MCT. Data obtained from this pilot study will provide guidance on how to target therapy based on racial differences in IR and BCF to improve clinical outcomes and racial

disparities in obesity and T2D. The physiology underlying racial differences in metabolism likely involves differences in BCF as well as in incretin response, leptin resistance, glucagon, variable tissue specific insulin sensitivity, and hepatic and extra-hepatic insulin clearance. The discovery of these hormonal pathways has already led to new therapies for T2D including GLP-1 receptor agonists and DPP4 inhibitors. However, the interactions between these pathways, race, and diet need to be better understood in order to lead to personalized treatments for metabolic diseases in the future.

Given the racially mixed patient population at BMC, we are uniquely qualified to conduct this cutting edge research designed to understand the metabolic basis of racial disparities and to seek new treatment paradigms for MS, T2D and obesity in the future.

3 STUDY DESIGN

This will be a six-week pilot clinical trial where MCT is administered orally to 24 subjects (approximately 12 AA, 12 CA).

Twenty-four subjects (12 AA, 12 CA) will participate in a pilot six-week clinical trial where MCT is administered orally. Subjects will be counseled to maintain their baseline body weight and physical activity level throughout the study. Dietary intake data will be obtained by the dietitian at the beginning of the study; individualized, weight-maintaining diet prescriptions will be based on resting energy expenditure (REE) measurements (via indirect calorimetry) and baseline physical activity levels (via International Physical Activity Questionnaire). Additional measurements include vitals, anthropometrics (height, weight, body mass index, and waist circumference), and body composition via dual energy x-ray absorptiometry (DXA). All measurements, except height, will be repeated at the end of the intervention period to measure any changes due to MCT.

Intervention. The dietary intervention will take place over 6 weeks (from Visits 3 through 9). Subjects will be instructed to take 30 grams MCT per 2000 kcal daily. The objective of this intervention is to replace approximately one-third of the subjects' dietary fat with MCT. Participants will accordingly reduce their intake of other sources of dietary fat, as prescribed by the study dietitian, to maintain a stable body weight during the trial.

At baseline and after the intervention, subjects will undergo assessment of insulin sensitivity by frequently sampled intravenous glucose tolerance test (FSIVGTT) to assess effects of study interventions on disposition index and BCF. Subjects are paid $200 for completing all study visits; they are paid $10 at Visit 1, $25 at Visit 2, $50 each at Visits 3 and 8 and $65 at Visit 9.

Timeline. We expect the last subject visit to be completed by the end of the grant period; therefore, all human samples will be collected by 12 months. In the subsequent 6 months, the study team will analyze collected data without need for further funding as well as begin preparing for an R01 application to further evaluate the metabolic effects of MCT and LCT in a larger blinded study.

Collaboration with the H-35166 Study Team. Both this study and H-35166 are performed by the same research team within the Section of Endocrinology, Diabetes, Nutrition and Weight Management. Additionally, H-35166 shares similar procedures with this protocol. Thus, subjects may be enrolled in both studies without duplicate procedures. In the instance that a subject qualifies for both studies, the study team will have access to data collected as part of an H-35166 study visit. The same is true for H-35166 having access to data collected through this study. This does not increase risk, since the research teams are comprised of the same individuals, all of whom have the required HIPAA and Human Subjects Training.

4 OUTCOME MEASURES

4.1 Primary outcome

The primary outcomes measures in this pilot study are change in insulin secretion and disposition index and change in insulin sensitivity.

4.1.1 Insulin Sensitivity and Bergman's Minimal Model analyses.

We have selected this model as the main method to measure Si in view of its extensive use in medical and epidemiologic studies, which would allow direct comparison with our data. Parameters of interest in this model include Si, the index of insulin sensitivity, insulin action expressed relative to "interstitial" insulin, glucose effectiveness (Sg) as a measure of the ability of glucose to enhance its own clearance, and the acute insulin response (AIR) as a measure of insulin secretion and BCF.

4.1.2 Disposition Index (DI) and Beta Cell Demand Index (BCDI).

The hyperbolic relationship between Si and BCF has been traditionally evaluated by the disposition index (DI)[22], which represents insulin secretion adjusted for Si (DI= AIR x Si). The BCDI is a quantitative measure of the "position" of AIR (a measure of BCF) data point on the hyperbola relative to the Si scale (BCDI = AIR/Si). Rightward shifts (higher Si) are associated with smaller slope (less demand) while leftward shifts along the hyperbola indicate more demand for β-cell secretion. Hepatic and extra-hepatic insulin clearance can be calculated from c-peptide kinetics[23].

4.2 Secondary outcomes

Secondary outcome measures include change in body weight and body composition.

4.2.1 Weight Change

Subjects will be instructed to follow an isocaloric diet designed to maintain body weight, however weight change can occur. Weight will be measured as designated in the Schedule of Events (Appendix 1).

4.2.2 Body Composition

Body composition will be determined by the gold standard method, DXA. This will allow us to determine the effects of MCT on both total lean mass and total fat mass.

4.3 Safety Measures

To ensure that the participant does not have any underlying health conditions, such as kidney or liver impairments, a blood chemistry panel and complete blood cell count will obtained during Screening. Vitals (blood pressure and pulse) will also be monitored as designated in the Schedule of Events (Appendix 1). A study physician will oversee each FSIVGTT. Study staff will monitor for adverse event during the 6-week intervention period; study physicians will be available for participant assessment as needed, as determined by the study staff. More information regarding safety measures can be found in Section 9 of the study protocol.

4.3.1 Pregnancy

For women of child bearing potential, a urine pregnancy test will be performed at Visit 1/Screening and prior to each DXA scan (Visits 2 and 8) and each FSIVGTT (Visits 3 and 9).

5 SUBJECT SELECTION

5.1 Number of Participants

We estimate that 24 participants will be needed to test the primary hypothesis. Taking into consideration a 50% screen fail rate, we ask for an upper limit sample size of 48 subjects.

5.2 Inclusion criteria

- English-speaking
- Males and female ambulatory subjects
- Self-identify as Caucasian/White or Black/African American
- Age ≥18 and ≤65 years
- BMI ≤45.0 kg/m^2

5.3 Exclusion criteria

- Diagnosis of type 2 diabetes or hemoglobin A1c >6.5
- Use of insulin, oral hypoglycemic, agents, or insulin-sensitizing agents
- Daily use of steroids
- Unstable weight within 3 months prior to baseline (e.g., weight gain or loss of >3%)
- Use of any weight loss medications or sex hormone therapy
- Daily use of psychotropic medications (for schizophrenia, bipolar disorder, obsessive compulsive disorder, posttraumatic stress disorder, psychotic disorder, mania)
- Chronic kidney disease, on dialysis or history of renal transplant
- Poorly controlled cardiovascular disease or congestive heart failure
- Severe peripheral vascular disease or severe liver disease
- Cancer
- A condition requiring use of oxygen such as severe COPD
- Women who are pregnant, lactating, or actively trying to become pregnant
- Any cognitive or other disorders that may interfere with participation or ability to follow restrictions
- Abnormal TSH levels (<0.01 or >1.5x the upper limit)
- Weight >450 lb (205 kg) or height > 6'6"
- Severe claustrophobia
- Has had or is preparing for bariatric surgery (pre- or post-bariatric)
- Medically required use of anticoagulant therapies
- Current use of MCT oil
- Anemia (hemoglobin and/or hematocrit outside sex-specific normal ranges)

5.4 Restrictions during the Course of the Study

The following restrictions apply during the study:

- Fast overnight for at least 12 hours (no food or beverage except water, and no more than one 8-oz. glass of water within 2 hours) prior to each visit where laboratory tests are performed
- Avoid alcohol and strenuous activity for 24 hours before each visit
- Avoid aspirin, NSAIDs, anticoagulants or other compounds (including omega-3 supplements and fish oil) that may affect bleeding, platelets or bruising for 72 hours before the frequently sampled IV glucose tolerance test (FSIVGTT)

- Inform the study team if doctor recommends any new prescription or over-the-counter medications
- Inform the study team if starting a diet or weight loss program (e.g., Weight Watchers®, Jenny Craig®) during the study

5.5 Participant Recruitment and Screening

Subjects will be recruited through the Nutrition and Weight Management Center at BMC by reviewing the medical records of patients with upcoming appointments. The study team will review the patient's sex, age, medical history, surgical history, ability to speak English, and body mass index (BMI) from EPIC. Potentially eligible subjects will be highlighted on a printout of the practitioner's schedule. Patients meeting the study criteria will be introduced to the study by a co-investigator during one of the patient's clinic visits. These patients will be screened in-person at the Nutrition and Weight Management Center using the Recruitment Script and Pre-Screen Form. Any interested participant can also choose to complete the prescreen form over the phone or by using the REDCap electronic survey.

Subjects will also be recruited through a variety of in-person, paper, and electronic methods. The study team will post the study on ResearchMatch.org, which is a national web-based recruitment registry that helps 'match' researchers with willing volunteers who may wish to participate in research studies. The study team will also recruit subjects via the Nutrition and Weight Management Center websites, BU and BMC all-campus weekly emails, Craigslist, and word-of-mouth. Additionally, current and previous Nutrition and Weight Management Center research subjects, who expressed an interest in participating in future studies, may be contacted if the signed informed consent form stated that they may be contacted. Subjects interested in H-35166 may also choose to pre-screen for H-35267 as well.

Interested subjects who contact the study team after their clinic appointments or from one of the aforementioned recruitment methods will be screened over the phone using the Recruitment Script and Pre-Screen Form. Identifiable information (name, address, email, phone number) will be recorded during the in-person or phone screening; this information will be recorded on the last sheet of the Pre-Screen Form. A list will be kept of the patients who fail this pre-screening process with their name and general reason for screen fail (i.e., medical history, BMI, age) so that non-eligible patients are not approached again. Identifiable information will be deleted from the list when the study recruitment is done, so that only anonymous screen fail reasons remain.

Those eligible for the study will be scheduled to meet with a research team member to obtain written informed consent. Subjects are required to give written informed consent prior to the completion of any study procedures.

6 STUDY PRODUCT

This intervention has been achieved by consuming a MCT-enriched cooking oil (Nestlé®, Glendale, CA). Each bottle contains 946 mL of MCT oil. This oil contains 7.7kcal/mL. Subjects will receive bottles of MCT oil to be used in drinks, salads and/or for all cooking needs over the 6-week study period. Dietary modifications will be provided on a per subject basis to maintain usual caloric intake. Subjects will have weekly contacts with the dietitian to re-enforce compliance, make dietary adjustments as needed, and for weight maintenance counseling.

6.1 Product Storage

Bulk supplies of study product will be stored in the Investigational Pharmacy Service (IPS) at controlled room temperature (20-25 Celsius). Study product will be labeled by the IPS staff for individual study participants prior to dispensing.

6.2 Blinding

This is an open-label study with no blinding. There is no randomization.

7 STUDY VISITS AND PROCEDURES

7.1 Screening

7.1.1 Pre-Screening

Potentially eligible participants will be identified from patient appointment logs in the Nutrition and Weight Management Center at Boston Medical Center as well as the electronic registry, ResearchMatch.org. Other research volunteers may approach the study team after seeing an electronic study advertisement (e.g., email, Nutrition and Weight Management Center websites, or Craigslist ad). Additionally, subjects who have screened for or are enrolled in H-35166 will be pre-screened to see if they might also qualify for this study. Patients who meet the preliminary study criteria (determined by completion of the Pre-Screen Form) and are interested in the study will be invited to complete Visit 1/Screening Visit.

7.1.2 Visit 1: Screening

Participants will first be asked to give written, informed consent. A screening visit will be completed to confirm subject eligibility. Assessments and procedures at the Screening Visit include:

- Informed consent
- Medical history
- Concomitant medications
- Demographics (self-reported)
- Vitals and anthropometrics (height, weight, blood pressure, pulse)
- Screening laboratory tests (fasting)
- Urine pregnancy test (for women of child-bearing potential)

Blood will be drawn to test for basic metabolic panel, complete blood count (CBC), TSH, hemoglobin A1c, and lipid panel. If any of these laboratory tests were performed in the 6 months prior to the screening visit at Boston Medical Center, they will not be redrawn and the values will be extracted from the medical record or previous Nutrition and Weight Management Center research documents. Alternatively, if the subject completed a screening visit for another study within the Nutrition and Weight Management Center, such as H-35166, within the last 6 months, the research team may elect to use those lab values. Lastly, if the potential subject has had any of the screening labs as part of routine medical care at an accredited institution and the subject chooses to share these results with the research team, they may also be used in place of new screening lab tests. Subjects will be paid $10 for completing Visit 1/Screening.

7.2 Visits 2 and 3: Baseline

Subjects who pass screening will complete a two-part Baseline Visit on separate days in the General Clinical Research Unit (GCRU). Subjects will complete the following procedures. Note that if the subject is also enrolled in H-35166, certain study procedures may not be duplicated.

- Visit 2:
  - Vitals and anthropometrics (blood pressure, pulse, weight, waist circumference)
  - Urine pregnancy test (for women of child-bearing potential)
  - Physical exam
  - Indirect calorimetry
  - iDXA scan
  - Questionnaire about physical activity
  - 24 hour food recall by dietitian

- Adverse event monitoring
- Subject payment ($25)

Visit 2 will occur within approximately 3 weeks of Visit 1/Screening. Due to the restricted scheduling availability of the investigators performing the FSIVGTT, Visit 2 may be delayed so as to maintain the approximate one week window between Visits 2 and 3.

- Visit 3:
  - Vitals and anthropometrics (blood pressure, pulse, weight)
  - Urine pregnancy test (for women of child-bearing potential)
  - Blood draw for banking*
  - Frequently sampled intravenous glucose tolerance test (FSIVGTT)
  - Dietary counseling
  - MCT oil dispensed
  - Adverse event monitoring
  - Subject payment ($50)

*Blood will be drawn at Visit 3/Baseline and banked for future testing of fructosamine, adiponectin, free fatty acids, ceramides, leptin, hs-CRP, fibrinogen, proinsulin, and other relevant tests; concurrent testing of these labs is not possible on the limited budget of this pilot study. Due to restricted scheduling availability, Visit 3 will occur within approximately one week of Visit 2.

7.3 Visits 4-7

Subjects will have weekly phone calls with the dietitian to reinforce compliance, make dietary adjustments as needed, and for weight maintenance counseling. Their dietary prescription may be modified as needed to maintain a stable weight during the study.

7.4 Visits 8 and 9: End-of-Intervention

At the end of the 6-week intervention, subjects will return for another two-part Post-Intervention Visit on separate days. Subjects will continue using the MCT oil until the day before Visit 9. Subjects will complete the following procedures:

- Visit 8:
  - Vitals and anthropometrics (blood pressure, pulse, weight, waist circumference)
  - Urine pregnancy test (for women of child-bearing potential)
  - Indirect calorimetry
  - iDXA scan
  - Questionnaire about physical activity
  - Adverse event monitoring
  - Subject payment ($50)

- Visit 9:
  - Vitals and anthropometrics (blood pressure, pulse, weight)
  - Urine pregnancy test (for women of child-bearing potential)
  - Blood draw for laboratory tests and banking*
  - Frequently sampled intravenous glucose tolerance test (FSIVGTT)
  - Remaining MCT oil collected
  - Adverse event monitoring
  - Subject payment ($65)

* At Visit 9, blood will be drawn to retest for lipid panel. Additional blood will be drawn and banked for future testing of fructosamine, adiponectin, free fatty acids, ceramides, leptin, hs-CRP, fibrinogen, proinsulin, and other relevant tests. Visit 9 will occur within approximately one week of Visit 8. Due to restricted scheduling availability, Visit 9 may be delayed so as to maintain the approximate one week window between Visits 8 and 9.

7.5 Study-specific Procedures

7.5.1 Medical history

Study staff will obtain a complete medical history. For women of non-child bearing potential, the reason should be documented.

7.5.2 Concomitant medications

During Screening and at each visit, all participants will be asked to provide the following information for any medication they are currently taking: 1) Drug Name (Generic name preferred), 2) Indication for Drug, 3) Frequency of Use, 4) Dosage level and Unit of Dose, 5) Route, and 6) Date Started. At subsequent study visits, all participants will be asked to report any new medications or changes to current medications. Changes can be made to the following fields: 1) Frequency of Use, 2) Dosage level and Unit of Dose, 3) Route, 4) Date Started, and 5) Date Stopped. When the participant completes the study, the final status of each active concomitant medication will be recorded. The study team will document whether a participant’s medication is ongoing at the end of the study.

7.5.3 Anthropometrics

7.5.3.1 Weight

For the measurement of body weight, participants must:

- Wear standard hospital-type gown or equivalent light clothing, with no shoes
- Have consumed no more than one 8-ounce glass of water within the 2 hours prior to measurement of body weight
- Void before measurement of body weight

Weight will be measured on a high capacity digital scale as designated in the Schedule of Events (Appendix 1). Study personnel will record the weight (in kg) to the first decimal point (e.g., 95.3 kg). The same scale will be used at each visit.

7.5.3.2 Height

Height is measured to the nearest 0.1 cm using a wall mounted stadiometer. Participants are instructed to: 1) remove shoes, 2) stand with back centered against the ruler, 3) place feet as close to the wall as possible with buttocks touching the wall, 4) keep eyes straight ahead even with ears, 5) make sure the chin is not lifted, 6) keep feet flat, 7) take a deep breath, exhale, take a maximum stretch, and stand tall. Height is measured to the nearest 0.1 cm applying firm pressure with stable marker to top of head centered with wall-mounted ruler.

7.5.3.3 Waist Circumference

For the measurement of waist circumference, the study-site personnel must ensure that:

- The participant stands and the examiner places a measuring tape in a horizontal plane around the abdomen at the level of the umbilicus or, in case of sagging abdomen, iliac crest.
- The measuring tape is snug, but does not compress the skin, is parallel to the floor, and is not twisted.

- The measurement is taken at the end of a normal respiratory expiration.
- The measurement (in cm) is recorded to the first decimal point.
- The anatomical landmark used when measuring waist circumference at Baseline (Week 1/Visit 2) should be noted, and the same landmark should be used for subsequent measurements.

7.5.4 Vitals

Systolic and diastolic blood pressure will be measured. Systolic and diastolic blood pressure should be measured after the participant rests for approximately 5 minutes and with the participant in a sitting position. Heart rate (pulse) will be measured over 1 minute after the blood pressure measurement has been completed and the result will be recorded.

7.5.5 Physical exam

A full physical examination (excluding pelvic and rectal examination) will be performed by the Investigator. The physical examination will cover the following areas: general appearance, eyes, ears, nose, throat, neck, respiratory, cardiovascular, endocrine, hepatic, gastrointestinal, peripheral vascular, neurological, musculoskeletal, extremities, lymphatic, dermatological, allergies and psychiatric.

7.5.5 Laboratory tests

To confirm eligibility, blood will be drawn at screening to test for basic metabolic panel, complete blood count (CBC), TSH, hemoglobin A1c, and lipid panel. If any of these laboratory tests were performed in the 6 months prior to the screening visit at Boston Medical Center, they will not be redrawn. The results will be extracted from the medical record, H-35166 source documents, or source documents of previous studies the subject has participated in through the Nutrition and Weight Management Center. If these blood tests have not been performed through routine care or another screening visit in the 6 months prior to screening, subjects will be asked to fast after midnight the night before testing and the tests will be performed by BMC phlebotomy and tested at the BMC Clinical Lab.

Blood will be drawn at Visits 3 and 9 and banked for future testing of fructosamine, adiponectin, free fatty acids, ceramides, leptin, hs-CRP, fibrinogen, proinsulin, and other relevant tests. At Visit 9, blood will also be drawn to retest lipid panel.

7.5.6 Indirect calorimetry

Indirect calorimetry will be used to measure resting energy expenditure. Patients will lay supine with their head under a transparent hood connected to a pump, which applies an adjustable ventilation through it. Exhaled gas is sampled. Ambient and diluted fractions of O_2 and CO_2 are measured for a known ventilation rate, and O_2 consumption and CO_2 production are determined and converted into Resting Energy Expenditure.

7.5.7 Physical Activity

Participants will complete the 2002 International Physical Activity Questionnaire at Visits 2 and 8. This consists of 27 questions about their level of physical activity in the previous 7 days. The questionnaire can be found in Appendix 2.

7.5.8 DXA Scan

Dual energy x-ray absorptiometry (DXA) scans will be performed in the General Clinical Research Unit at Boston Medical Center. The scan will be performed on a Lunar iDXA Machine. Participants are asked to remove all external metal objects including jewelry and undergarments containing

metal and then clothed in a light hospital gown. They are asked to lie down on the scanning table with their arms at their sides. A certified technician performs each 6-minute scan. A full results report is printed for each participant and added to the study source documents. Regional differences in adipose tissue thickness and distribution (upper vs. lower body fat) will be calculated from the scan data.

7.5.9 Urine Pregnancy Test

A negative pregnancy test is required for all women of child bearing potential at Screening and prior to each DXA scan and FSIVGTT. Women who have had their uterus removed (hysterectomy), both of their ovaries removed (bilateral oophorectomy), or who have undergone a bilateral tubal ligation are considered not of child bearing potential.

7.5.10 Frequently Sampled Intravenous Glucose Tolerance Test

The Frequently Sampled Intravenous Glucose Tolerance Test (FSIVGTT) will be conducted in the GCRU under direct supervision by Drs. Istfan or Apovian. Subjects are asked to fast after midnight the night prior to the procedure. The intravenous glucose dose will be prepared in advance by the BMC Investigational Pharmacy Service as a 20% glucose solution. The volume to be administered will be calculated according the subject's body weight, so as to give 300 mg glucose per kilogram body weight. The body weight used for the calculation will be taken from the previous visit (i.e, Visit 2 weight used for Visit 3 FSIVGTT and Visit 8 weight used for Visit 9 FSIVGTT). The pharmacy will provide the exact volume for each subject in a sterile infusion bag (usual volume 90 to 180 ml). The procedure is started by placing an 18-gauge catheter in an antecubital vein of each arm. The catheter used for sampling (but not for infusion of the glucose) will be connected to a 3-way stop-cock mechanism for frequent blood sampling. Vein patency is maintained by saline infusion at a rate of 20 ml/min.

Baseline blood is drawn at -20, -15, -10 and -5 minutes prior to the administration of the glucose bolus dose. The glucose bolus is administered by rapid intravenous drip (wide open catheter line, usually over 2-3 minutes). Time=0 is defined as the time when the infusion of glucose into the arm/catheter not used for sampling is completed. At the following times, 3-ml blood samples will be drawn: 1, 2, 3, 4, 5, 6, 8, 10, 12, 14, 16 and 19 minutes. At 20 minutes, a bolus of insulin (Humulin® R) is given, calculated as 0.025 units per kg body weight. As with the glucose solution, the body weight used for the calculation will be taken from the previous visit. Blood is then drawn at the following times: 22, 25, 30, 40, 50, 60, 70, 80, 90, 100, 110, 120, 140, 160 and 180 min. The blood samples are divided into 1 ml (glucose) and 2 ml (insulin) portions. Additional 3-ml samples will be collected with a syringe at -5, 5, 15, 30, 60 and 120 min (up to 20 ml total) for measurement of ROS (reactive oxygen species) which requires specific, immediate processing. The total amount of blood to be collected is approximately 120 ml. Subjects will be given a small snack at the termination of the procedure.

If a subject is also enrolled in H-35166, the Visit 3 FSIVGTT may not be duplicated.

7.5.11 Dietary Counseling

All subjects will be counseled to maintain their baseline body weight and physical activity level throughout the study. Dietary intake data will be obtained by the dietitian at the beginning of the study at Visit 2; individualized, weight- maintaining diet prescriptions will be based on resting energy expenditure (REE) measurements (via indirect calorimetry) and baseline physical activity levels (via International Physical Activity Questionnaire). Dietary prescription will be given by the study dietitian with the goal of minimally altering the baseline macronutrient composition of the participants' diets.

Throughout the intervention period, the dietitian will have weekly phone calls with the subjects to reinforce compliance, make dietary adjustments as needed, and for weight maintenance counseling.

7.5.12 Dispensing Study Product

The study product, MCT oil, (Nestlé, Glendale, CA) will be dispensed by the BMC Investigational Pharmacy Service (IPS). Each subject will receive enough MCT oil to last the entire 6-week intervention period (from Visit 3 until Visit 9). The MCT oil will be labeled by the IPS staff for individual study participants prior to dispensing and dispensed in the unopened amber glass bottles in which it is received by Nestlé. Total energy expenditure (TEE) per subject will be calculated based on resting energy expenditure (REE) x physical activity (PAL) from the IPAQ questionnaire. The tables below provide approximate calculations with hypothetical subject data.

Subject #	Subject age (years)	PAL	Visit 2 weight (kg)	Height (cm)	BMI (kg/m^2)	REE (kcal)	TEE (kcal)
1	36	1.00	79.5	163.0	29.9	2000	2000
2	36	1.54	103.0	160.0	40.2	2000	3080

Subject #	Daily MCT oil dose (grams)[a]	Daily calories from MCT oil (kcals)[b]
1	30	249
2	46	382

[a]Daily grams of MCT calculated by TEE/2000 kcals * 30g.
[b]Daily calories from MCT = daily g MCT * 8.3 kcal/g

Subject #	Weekly MCT oil dose (grams)[a]	Weekly calories from MCT oil (kcals)[a]
1	210	1743
2	323	2674

[a]Weekly dose of MCT oil is calculated by multiplying the daily dose by 7.

Subject #	Total MCT oil dose (grams)[a]	Total calories from MCT oil (kcal)[a]	# bottles MCT oil needed (bottles)
1	1260	10458	1.3
2	1940	16044	2.1

[a]Total dose of MCT oil for the entire 6 week intervention is calculated by multiplying the weekly dose by 6. This total dose will show the maximum of number of bottles of MCT oil needed per subject.

We estimate that no subject will need more than 3 bottles for the entire 6-week intervention period. If a subject breaks or loses a bottle of MCT oil, they will be asked to contact the study site and the bottle will be replaced by IPS.

7.5.13 Adverse Event Monitoring

All AEs and SAEs will be recorded (see Section 9)

7.5.14 Product Accountability

During each weekly contact with the dietitian, the subject will be asked about their daily compliance. At Visit 3, MCT oil bottles will be weighed and recorded to the nearest 0.1 g. At Visit 9, the subject will return all dispensed bottles to the study site. The study staff will measure the weight of all remaining oil and calculate the percent compliance of oil consumed as outlined below.

Total weight dispensed (g)	-	Total weight prescribed by dietitian (g)	=	Expected amount consumed (g)

Expected weight consumed (g)	÷	Actual weight returned by subject (g)	X 100	=	% compliance

7.5.15 Subject Payments

Subjects will be paid per the following schedule. If a subject is also enrolled in H-35166 and performs a procedure that is included in both studies (specifically, the FSIVGTT), they will only be paid by one study. For example, Visit 3 includes nearly identical procedures to H-35166's Baseline Day 1 study visit. Thus, the subject will be paid only one payment of $50 for Visit 3, rather than being paid by both studies.

**Visit Name**	**Amount**
Visit 1/Screening	$10
Visit 2	$25
Visit 3	$50
Visit 8	$50
Visit 9	$65
Total for all visits	$200

Payments will be pro-rated as subjects complete each visit.

7.5.16 Medical Record Review

Medical record review and/or release may be requested of the subjects in several instances.

The study team may review the subject's medical records in order to ascertain routinely collected information such as laboratory test results. This would minimize risk and burden to the subject by potentially allowing the subject to forego the screening laboratory tests.

Additionally, for subjects who have previously completed a study screening visit through the Nutrition and Weight Management Center at BMC, the study team may review previously collected laboratory tests in an effort to minimize the risk and burden of screening labs on the participant.

Medical records may also be requested in the case that a subject is hospitalized during this study.

8 STATISTICAL PLAN

8.1 Data Analysis

We will use multivariable statistical models, adjusted for potential changes in body weight and composition, REE, and physical activity level to 1) control for potential modifiers of insulin sensitivity and insulin secretion, separately from the effect of race; 2) determine the relationship between MCT, race, and minimal model parameters and insulin clearance. Analysis will be done by ANOVA (Analysis of Variance). Statistically significant difference is defined by P =< 0.05. All data will be presented in mean ± SE.

8.2 Sample Size Justification

Because this study serves as preliminary pilot study to gather data to justify a larger study, 24 subjects will provide adequate power to be able to prove the hypothesis. Taking into consideration a 50% screen fail rate we will recruit up to 48 subjects.

9 SAFETY MONITORING AND ADVERSE EVENTS

9.1 Definitions

Definitions are per the January 2007 Guidance on Reviewing and Reporting Unanticipated Problems Involving Risks to Participants or Others and Adverse Events, Office on Human Research Protection (OHRP) Guidance. http://www.hhs.gov/ohrp/policy/AdvEvntGuid.htm.

9.1.1 Adverse Event

An *adverse event (AE)* is any untoward or unfavorable medical occurrence in a human study participant, including any abnormal sign (for example, abnormal physical exam or laboratory finding), symptom, or disease, temporally associated with the participant's involvement in the research, whether or not considered related to the participant's participation in the research.

9.1.2 Serious Adverse Event

A *serious adverse event* (SAE*)* is any AE that is:

- fatal
- life-threatening
- requires or prolongs hospital stay
- results in persistent or significant disability or incapacity
- results in congenital anomalies or birth defects
- an important medical event*

Important medical events* are those that may not be immediately life threatening, but are clearly of major clinical significance.

9.1.3 Unanticipated Problem

An Unanticipated Problem is any incident, experience, or outcome that meets all of the following criteria:

- unexpected (in terms of nature, severity, or frequency) given the research procedures that are described in the IRB-approved research protocol and informed consent document;
- related or possibly related to participation in the research; possible related means that there is a reasonable possibility that the incident, experience or outcome may have been caused by the procedures involved in the research.
- suggests that the research places participants or others at a greater risk of harm (including physical, psychological, economic, or social harm) related to the research than was previously known or recognized.

9.2 Adverse Event Reporting Period

The study period during which adverse events must be reported is normally defined as the period from the initiation of any study procedures to the end of the study intervention follow-up.

9.3 Preexisting Condition

A preexisting condition is one that is present at the time of signing the consent form for the main study. A preexisting condition should be recorded as an adverse event if the frequency, intensity, or the character of the condition worsens during the study period.

9.4 Post-study Adverse Event

All unresolved adverse events should be followed by the investigator until the events are resolved, the participant is lost to follow-up, or the adverse event is otherwise explained. At the last scheduled visit, the investigator should instruct each participant to report any subsequent event(s) that the participant, or the participant's personal physician, believes might reasonably be related to participation in this study. Due to the nature and composition of the study products, no delayed toxicities or withdrawal effects are expected after a participant has discontinued participation in the study. Therefore, no collection of safety information will be done after participant's discontinuation from the study.

9.5 Abnormal Laboratory Values

A clinical laboratory abnormality should be documented as an adverse event if any one of the following conditions is met:

- The laboratory abnormality is not otherwise refuted by a repeat test to confirm the abnormality
- The abnormality suggests a disease and/or organ toxicity
- The abnormality is of a degree that requires active management

9.6 Hospitalization, Prolonged Hospitalization or Surgery

Any adverse event that results in hospitalization should be documented and reported as a serious adverse event. Any condition responsible for surgery should be documented as an adverse event if the condition meets the criteria for an adverse event and reported as a severe adverse event if hospitalization is required. Neither the condition, hospitalization, nor surgery is reported as an adverse event if the hospitalization was for diagnostic or elective surgical procedures for a preexisting condition.

9.7 Pregnancy

For women of child-bearing potential, a urine pregnancy test will be performed at Visit 1/Screening and prior to each DXA scan and FSIVGTT unless participant is surgical sterile (documented hysterectomy, bilateral oophorectomy, or bilateral tubal ligation). Participants who become pregnant during the study will not receive any additional study product and will be withdrawn from the study immediately upon confirmation of pregnancy. The reason for withdrawal will be documented on the disposition CRF page as a "protocol violation." These participants will be required to return to the clinical study site for early termination procedures depending on last visit completed. Documented pregnancies will not be monitored through outcome.

The risk of pregnancy is minimized by advising that all women of child-bearing potential agree to use one of the acceptable methods of contraception or be abstinent during the study. Acceptable methods include each of the following: male or female condom, diaphragm or cervical cap, combination or progestin-only birth control pills (oral contraceptives), vaginal contraceptive rings, contraceptive patches, intrauterine devices, Depo-Provera (stable for 6 months), and implantable contraceptives.

9.8 Recording of Adverse Events

At each contact with the participant, the investigator or study staff will seek information on adverse events by specific questioning and, as appropriate, by examination. Information on all adverse events should be recorded in the source document, and also in the appropriate adverse event module of the CRF. All clearly related signs, symptoms, and abnormal diagnostic procedures

results should be recorded in the source document. All adverse events occurring during the study period must be recorded.

Because research is separate from medical care, the PI or co-investigator (co-I) may contact the subject in the case of an abnormal laboratory value, at which point the PI or co-I will refer the patient to their primary care physician for routine care. In these cases, a signed progress note will be filed with the participant's source documents. Additionally, the subject may consent for the investigator(s) to mail a letter to their primary care physician. The clinical course of each event should be followed until resolution, stabilization, or until it has been determined that the study intervention or participation is not the cause. Serious adverse events that are still ongoing at the end of the study period must be followed up to determine the final outcome.

We expect MCT supplementation to be well tolerated by subjects. Adverse effects will be monitored via weekly contacts. The procedures proposed in the current application have all been extensively used and validated.

9.9 Potential Risks to Participants

The potential risks of the study include the risks of blood drawing, medium chain triglycerides, DXA scan and the administration of questionnaires.

9.9.1 Blood Draws

The risks of blood drawing include pain, bleeding, and/or a bruise at the site of needle insertion. Serious complications such as blood clots or infection are very rare when proper precautions are taken.

9.9.2 MCT Oil

Adverse effects that are commonly described with ingestion of MCT oil are nausea, vomiting, bloating, emesis, gastrointestinal discomfort, abdominal cramps, and osmotic diarrhea. These symptoms are minimal when MCT oil is consumed together with other meal components, as it will be in this study. To prevent these possible side effects, it will be recommended that no more than 15ml MCT oil be ingested at one time. If any symptoms develop as a result of the MCT oil, subjects will be instructed at Visit 3 (when product is dispensed) to contact the study dietitian to discuss reducing their daily MCT intake temporarily until they resolve. The majority of side effects can be prevented with proper hydration and full product compliance. In addition, some studies suggest a possibility of hypertriglyceridemia from consumption of MCT oil.

9.9.3 DXA Scans

The radiation exposure during a DXA scan is minimal (about one tenth the amount experienced when getting a chest x-ray). While we do not know whether any dose of radiation is completely safe, this amount of radiation to which the participants will be exposed during the course of the study is well within the limits considered "safe" by federal and state regulations.

9.9.4 Questionnaire

The instruments used for the assessment of self-reported physical activity ask about personal details that the participants might find embarrassing. Although we encourage participants to answer all the questions, they have the option of not answering the questions that they might find embarrassing. All the questionnaires are coded and confidentiality is maintained at all times.

9.9.5 FSIVGTT

Low blood glucose levels and symptomatic hypoglycemia are commonly experienced by the subjects due to rapid fall of glucose concentration after administration of the insulin bolus at minute 20 of the test. This is part of the test and is needed for improved accuracy of the calculation of insulin sensitivity index by minimal model analysis. Hypoglycemia is expected between 60-90 minutes of the test, lasting not more than 15 minutes. The average duration of symptoms experienced by the subjects is 5-10 minutes and usually consist of sweating, nausea, dizziness and light headedness. These symptoms subside spontaneously as blood sugar starts to stabilize after 90 minutes. Termination of the test because of these symptoms is very rare. Reasons for termination include request by the subject; prolonged symptoms (more than 15 minutes); drop in blood pressure (below 90 systolic). Blood glucose levels are monitored during the procedure. Levels below 50 mg/dl, lasting longer than 15 minutes, will lead to termination of the test. If that occurs, 8 oz. of carbohydrate will be administered to raise the blood glucose. There is also a small risk of phlebitis (inflammation or infection of the veins) from placement of the IV catheter.

9.10 Procedures for Protecting Against Potential Risks

All safety related information will be collected and processed promptly, to comply with regulatory requirements designed to protect study subjects.

9.10.1 Blood draws

Blood will be drawn by trained hospital phlebotomists. No more than 10 mL of blood will be collected at any visit. If a subject feels faint after a blood draw, they will lie down right away to avoid falling down.

9.10.2 MCT Oil

To prevent possible side effects related to MCT oil, it will be recommended that no more than 15ml MCT oil be ingested at one time. Additionally, subjects will be instructed to consume MCT oil with other meal components. We will also closely monitor patients and advise them to call the investigators for any adverse events. The study will be stopped for any subject experiencing serious side effects.

9.10.3 DXA

There is only minimal risk to an iDXA procedure. This scan is performed by a trained professional.

9.10.4 FSIVGTT

Risk is minimized by having experienced professionals perform these procedures. The procedures are performed in the GCRU where the subjects are closely monitored by a study clinician or nurse.

9.10.5 Therapeutic Misconception

Potential candidates will not be consented by their provider to help reduce any undue coercion. Further, they will be informed that any decision they make regarding their study participation will not affect the healthcare they are receiving. This information will be covered during the screening/consent process.

9.10.6 Breach of Confidentiality

Only the PI, investigators and study personnel on this project will have access to the master code. In addition, the investigators will be using password-protected computers and locked cabinets for data and sample storage, respectively.

9.11 Data and Safety Monitoring Plan

Per the OHRP Guidelines, this study is defined as “greater than minimal risk” or “low” risk therefore we will not establish a Data Safety and Monitoring Board (DSMB). However, study investigators will meet on a monthly basis to review study data and reported adverse events. In addition to the procedures outlined above, data on potential adverse events will be collected as designated in the Schedule of Events (Appendix 1). Participants will also be asked about recent hospitalizations and illnesses.

The PI will uphold the following responsibilities:

- The Principal Investigator (PI) and members of the staff will be responsible for reporting all new clinical experiences, exacerbations, and/or deterioration of any existing clinical condition occurring after a study subject has entered the study.
- The PI will be responsible for reporting Serious Adverse Events (SAEs) and Unanticipated Problems (UPs) to the BMC IRB. Per OHRP Guidelines all UPs should be reported to appropriate institutional officials (as required by an institutions written reporting procedures), the supporting agency head (or designee), and OHRP within one month of the IRB’s receipt of the report of the problem from the investigator.
- The PI will be responsible for determining the causality of all AEs/SAEs subject to review by the BMC IRB.
- The PI will be responsible for determining whether test results are “clinically significant” (CS) or “not clinically significant” (NCS). The PI will sign and date all lab reports as “CS” or “NCS”.
- The PI and staff will be responsible for follow-up information on all AEs until resolution or an appropriate endpoint is reached.

The PI in concert with BMC IRB and other study investigators will be responsible for reviewing all reports of SAEs that occur in this study and for determining whether any corrective actions need to be taken regarding: management decisions of the PI and staff, whether protocol violations are congruous with patient welfare taking precedence over protocol, whether there are any issues among the research staff that need to be addressed, etc. The study investigators will be responsible to determine if the results of its review require a revision/modification of the RPN and/or the consent form. There will be no independent reviewer may be considered if there is potential for conflict of interest.

If any expected event listed in the consent form/protocol exceeds what it expected in terms of incidence or severity and is thought to be related to study procedures, the study will stop any procedures until more can be known about the cause of the excess.

10 DATA MANAGEMENT

10.1 General Guidelines

The site will perform all data management activities, including the writing of a data management plan outlining the systems and procedures to be used.

Clinical study data will be reported (captured) by study site personnel on paper source documents then transcribed into an electronic database. The electronic data will be entered by two separate study site staff members. The two databases will then be compared on a biweekly basis for discrepancies using a SPSS program. All discrepancies will be corrected and the electronic data will be reviewed by the PI or their designee monthly. For data analysis, all data will be downloaded from the electronic databases and reformatted into SPSS data sets.

10.2 Discontinuation of Participants

Every effort should be made to conduct all protocol-required procedures to complete the study. Participants may be removed from the study for the following reasons:

- **Withdrawal of Consent:** Participant wishes to exercise the right to withdraw from the study as stated in the ICF (all participants reserve the right to withdraw from the study without prejudice).
- **Adverse Event:** Participant experiences an adverse event that, in the investigator's opinion, necessitates withdrawal from the study.
- **Investigator Decision:** Investigator feels it is in the participant's best interest to terminate participation for reasons other than an adverse event.
- **Protocol Violation:** Participant is noncompliant with protocol procedures, becomes pregnant, violates study entry criteria, or starts an exclusionary concomitant medication.
- **Lost to Follow-Up:** Participant fails to return for study visits and cannot be reached with reasonable, repeated attempts.
- **Administrative Reason:** The study sponsor or other regulatory authority discontinues the study protocol or the clinical study site discontinues participation.

Any withdrawal must be fully documented in the participant's source records and recorded on the disposition page of the CRF. The documentation must include the reason for the withdrawal and details of any sequelae (followed until symptoms resolve or improve, as appropriate). Withdrawals due to an adverse event must be documented on both the disposition page and the adverse event page of the CRF.

When a participant is lost to follow-up (i.e., fails to return for study visits), a reasonable effort (e.g., documented by receipts for certified mailings) will be made to contact the participant to determine why the participant failed to return and to attempt to schedule the Early Termination visit.

11 ADMINISTRATIVE PROCEDURES

11.1 Compliance with the Protocol and Protocol Revisions

The study shall be conducted as described in this approved protocol. Deviations or changes to the protocol shall be made only after amendment approval/favorable opinion by the IRB, except where necessary to eliminate immediate hazard to study participants. Any significant deviation will be documented in the study team progress notes.

If an amendment substantially alters the study design or increases potential risk for study participants, the Informed Consent should be revised and submitted to the IRB. Once the IRB has approved the revised Informed Consent, all study participants currently enrolled in the study must sign the amended consent, and the new consent will be used for all new participants prior to enrollment.

11.2 Informed Consent

Study investigators must ensure that participants or their legally acceptable representative are clearly and fully informed about the purpose, potential risks and other critical issues regarding this clinical trial.

The preparation of the Informed Consents used for this study is the responsibility of the PI and must include all elements required by ICH, GCP and applicable regulatory requirements. Prior to beginning the study, the Informed Consents will be approved by the IRB. The consent forms used will bear the IRB stamp on all pages.

11.3 Informed Consent Procedures

The consenting procedure used for this study complies with GCP. All eligible and willing volunteers will be consented prior to any procedures being performed. The consent process will occur in a private area. Subjects will be given opportunity prior to the consent process to read and review the consent form. During the informed consent process, study staff will review main areas of the study design, address all subject concerns, reinforce the study's commitment to the subject and reinforce the commitment needed from the subject to be in the study. Subjects will be allowed as much time as they need prior to signing the consent form. If the subject still has concerns that the study staff cannot address, they will be provided the opportunity to speak with one of the investigators. The subject will be given a photocopy of the completed, signed consent form.

Informed consent will not be obtained by the subject's clinical provider.

11.4 Participants Unable to Give Informed Consent

Participants who are not able to give informed consent will not be considered for this study.

11.5 Records and Reports

The PI is required to prepare and maintain adequate and accurate case histories for each participant involved in the study. Source documents and electronic records entered in the study database must be consistent with each other, and discrepancies explained.

Corrections must be made by striking incorrect data with single line and entering correct information followed by authorized person’s initials and date.

The information in source documents must be reviewed, signed and dated by the PI or a Sub-Investigator.

11.6 Institutional Review Board (IRB)

Before study initiation, the PI must have written and dated approval/favorable opinion from an IRB for the protocol, consent form/s, participant recruitment materials, and any other written information provided to participants. The PI should also provide the IRB with an Investigational Brochure or product labeling.

The PI will provide the IRB with reports, updates and any other information according to regulatory requirements or IRB procedures.

11.7 Record Retention

The PI will retain investigational product disposition records, electronic database files, and source documents for a minimum of 3 years after completion of the study. The database master code list will be deleted after completion of the study, though de-identified data will be maintained.

APPENDIX 1. SCHEDULE OF EVENTS

	Screening (≤21 days)	Baseline		Open-label intervention					End of Intervention
**Week**	**-3**	**-1**	**0**	**1**	**2**	**3**	**4**	**6**	**7**
**Visit Number**	**1**	**2**	**3**	**4**	**5**	**6**	**7**	**8**	**9**
**Study Procedure**									
Informed consent	X								
Medical history	X								
Concomitant medications	X								
Height	X								
Weight, Body mass index	X	X	X					X	X
Vitals (blood pressure, pulse)	X	X	X					X	X
Laboratory tests	X[a]		X[b]						X[c]
Indirect calorimetry		X						X	
International Physical Activity Questionnaire		X						X	
Physical exam		X							
Frequently-sampled intravenous glucose tolerance test			X						X
Waist circumference		X						X	
Dietitian visit			X	X	X	X	X	X	
Dispense product			X						
DXA scan		X						X	
Product accountability				[d]	[d]	[d]	[d]	[d]	X
Urine pregnancy test[e]	X	X	X					X	X
Adverse event monitoring		X	X	X	X	X	X	X	X
Issue study voucher[f]	X	X	X					X	X

[a] Laboratory tests for eligibility will only be performed if blood tests have not been performed in clinic or as part of screening procedures for studies within the Nutrition and Weight Management Center within the past 6 months.
[b] At visits 3 and 9, blood will be banked for future testing.
[c] At visit 9, blood will be drawn to retest lipid panel post-intervention and banked for future testing.
[d] Compliance with the MCT oil prescription will be assessed and reinforced by the dietitian through weekly contacts (Visits 4 through 7).
[e] Urine pregnancy tests will be performed for all subjects of childbearing potential. If a subject is not of childbearing potential, the reason will be documented in the source documents.
[f] Subjects are paid $10 at Visit 1, $25 at Visit 2 and $50 each at Visits 3 and 8 and $65 at Visit 9. Total is $200. Payments are pro-rated.

APPENDIX 2. INTERNATIONAL PHYSICAL ACTIVITY QUESTIONNAIRE

INTERNATIONAL PHYSICAL ACTIVITY QUESTIONNAIRE (October 2002)

LONG LAST 7 DAYS SELF-ADMINISTERED FORMAT

FOR USE WITH YOUNG AND MIDDLE-AGED ADULTS (15-69 years)

The International Physical Activity Questionnaires (IPAQ) comprises a set of 4 questionnaires. Long (5 activity domains asked independently) and short (4 generic items) versions for use by either telephone or self-administered methods are available. The purpose of the questionnaires is to provide common instruments that can be used to obtain internationally comparable data on health–related physical activity.

Background on IPAQ
The development of an international measure for physical activity commenced in Geneva in 1998 and was followed by extensive reliability and validity testing undertaken across 12 countries (14 sites) during 2000. The final results suggest that these measures have acceptable measurement properties for use in many settings and in different languages, and are suitable for national population-based prevalence studies of participation in physical activity.

Using IPAQ
Use of the IPAQ instruments for monitoring and research purposes is encouraged. It is recommended that no changes be made to the order or wording of the questions as this will affect the psychometric properties of the instruments.

Translation from English and Cultural Adaptation
Translation from English is encouraged to facilitate worldwide use of IPAQ. Information on the availability of IPAQ in different languages can be obtained at www.ipaq.ki.se. If a new translation is undertaken we highly recommend using the prescribed back translation methods available on the IPAQ website. If possible please consider making your translated version of IPAQ available to others by contributing it to the IPAQ website. Further details on translation and cultural adaptation can be downloaded from the website.

Further Developments of IPAQ
International collaboration on IPAQ is on-going and an ***International Physical Activity Prevalence Study*** is in progress. For further information see the IPAQ website.

More Information
More detailed information on the IPAQ process and the research methods used in the development of IPAQ instruments is available at www.ipaq.ki.se and Booth, M.L. (2000). *Assessment of Physical Activity: An International Perspective.* Research Quarterly for Exercise and Sport, 71 (2): s114-20. Other scientific publications and presentations on the use of IPAQ are summarized on the website.

INTERNATIONAL PHYSICAL ACTIVITY QUESTIONNAIRE

We are interested in finding out about the kinds of physical activities that people do as part of their everyday lives. The questions will ask you about the time you spent being physically active in the **last 7 days**. Please answer each question even if you do not consider yourself to be an active person. Please think about the activities you do at work, as part of your house and yard work, to get from place to place, and in your spare time for recreation, exercise or sport.

Think about all the **vigorous** and **moderate** activities that you did in the **last 7 days**. **Vigorous** physical activities refer to activities that take hard physical effort and make you breathe much harder than normal. **Moderate** activities refer to activities that take moderate physical effort and make you breathe somewhat harder than normal.

PART 1: JOB-RELATED PHYSICAL ACTIVITY

The first section is about your work. This includes paid jobs, farming, volunteer work, course work, and any other unpaid work that you did outside your home. Do not include unpaid work you might do around your home, like housework, yard work, general maintenance, and caring for your family. These are asked in Part 3.

1. Do you currently have a job or do any unpaid work outside your home?

☐ Yes

☐ No → ***Skip to PART 2: TRANSPORTATION***

The next questions are about all the physical activity you did in the **last 7 days** as part of your paid or unpaid work. This does not include traveling to and from work.

2. During the **last 7 days**, on how many days did you do **vigorous** physical activities like heavy lifting, digging, heavy construction, or climbing up stairs **as part of your work**? Think about only those physical activities that you did for at least 10 minutes at a time.

_____ **days per week**

☐ No vigorous job-related physical activity → ***Skip to question 4***

3. How much time did you usually spend on one of those days doing **vigorous** physical activities as part of your work?

_____ **hours per day**
_____ **minutes per day**

4. Again, think about only those physical activities that you did for at least 10 minutes at a time. During the **last 7 days**, on how many days did you do **moderate** physical activities like carrying light loads **as part of your work**? Please do not include walking.

_____ **days per week**

☐ No moderate job-related physical activity → ***Skip to question 6***

LONG LAST 7 DAYS SELF-ADMINISTERED version of the IPAQ. Revised October 2002.

5. How much time did you usually spend on one of those days doing **moderate** physical activities as part of your work?

_____ **hours per day**
_____ **minutes per day**

6. During the **last 7 days**, on how many days did you **walk** for at least 10 minutes at a time **as part of your work**? Please do not count any walking you did to travel to or from work.

_____ **days per week**

☐ No job-related walking → ***Skip to PART 2: TRANSPORTATION***

7. How much time did you usually spend on one of those days **walking** as part of your work?

_____ **hours per day**
_____ **minutes per day**

PART 2: TRANSPORTATION PHYSICAL ACTIVITY

These questions are about how you traveled from place to place, including to places like work, stores, movies, and so on.

8. During the **last 7 days**, on how many days did you **travel in a motor vehicle** like a train, bus, car, or tram?

_____ **days per week**

☐ No traveling in a motor vehicle → ***Skip to question 10***

9. How much time did you usually spend on one of those days **traveling** in a train, bus, car, tram, or other kind of motor vehicle?

_____ **hours per day**
_____ **minutes per day**

Now think only about the **bicycling** and **walking** you might have done to travel to and from work, to do errands, or to go from place to place.

10. During the **last 7 days**, on how many days did you **bicycle** for at least 10 minutes at a time to go **from place to place**?

_____ **days per week**

☐ No bicycling from place to place → ***Skip to question 12***

LONG LAST 7 DAYS SELF-ADMINISTERED version of the IPAQ. Revised October 2002.

11. How much time did you usually spend on one of those days to **bicycle** from place to place?

_____ **hours per day**
_____ **minutes per day**

12. During the **last 7 days**, on how many days did you **walk** for at least 10 minutes at a time to go **from place to place**?

_____ **days per week**

☐ No walking from place to place → ***Skip to PART 3: HOUSEWORK, HOUSE MAINTENANCE, AND CARING FOR FAMILY***

13. How much time did you usually spend on one of those days **walking** from place to place?

_____ **hours per day**
_____ **minutes per day**

PART 3: HOUSEWORK, HOUSE MAINTENANCE, AND CARING FOR FAMILY

This section is about some of the physical activities you might have done in the **last 7 days** in and around your home, like housework, gardening, yard work, general maintenance work, and caring for your family.

14. Think about only those physical activities that you did for at least 10 minutes at a time. During the **last 7 days**, on how many days did you do **vigorous** physical activities like heavy lifting, chopping wood, shoveling snow, or digging **in the garden or yard**?

_____ **days per week**

☐ No vigorous activity in garden or yard → ***Skip to question 16***

15. How much time did you usually spend on one of those days doing **vigorous** physical activities in the garden or yard?

_____ **hours per day**
_____ **minutes per day**

16. Again, think about only those physical activities that you did for at least 10 minutes at a time. During the **last 7 days**, on how many days did you do **moderate** activities like carrying light loads, sweeping, washing windows, and raking **in the garden or yard**?

_____ **days per week**

☐ No moderate activity in garden or yard → ***Skip to question 18***

LONG LAST 7 DAYS SELF-ADMINISTERED version of the IPAQ. Revised October 2002.

17. How much time did you usually spend on one of those days doing **moderate** physical activities in the garden or yard?

_____ **hours per day**
_____ **minutes per day**

18. Once again, think about only those physical activities that you did for at least 10 minutes at a time. During the **last 7 days**, on how many days did you do **moderate** activities like carrying light loads, washing windows, scrubbing floors and sweeping **inside your home**?

_____ **days per week**

☐ No moderate activity inside home → ***Skip to PART 4: RECREATION, SPORT AND LEISURE-TIME PHYSICAL ACTIVITY***

19. How much time did you usually spend on one of those days doing **moderate** physical activities inside your home?

_____ **hours per day**
_____ **minutes per day**

PART 4: RECREATION, SPORT, AND LEISURE-TIME PHYSICAL ACTIVITY

This section is about all the physical activities that you did in the **last 7 days** solely for recreation, sport, exercise or leisure. Please do not include any activities you have already mentioned.

20. Not counting any walking you have already mentioned, during the **last 7 days**, on how many days did you **walk** for at least 10 minutes at a time **in your leisure time**?

_____ **days per week**

☐ No walking in leisure time → ***Skip to question 22***

21. How much time did you usually spend on one of those days **walking** in your leisure time?

_____ **hours per day**
_____ **minutes per day**

22. Think about only those physical activities that you did for at least 10 minutes at a time. During the **last 7 days**, on how many days did you do **vigorous** physical activities like aerobics, running, fast bicycling, or fast swimming **in your leisure time**?

_____ **days per week**

☐ No vigorous activity in leisure time → ***Skip to question 24***

LONG LAST 7 DAYS SELF-ADMINISTERED version of the IPAQ. Revised October 2002.

23. How much time did you usually spend on one of those days doing **vigorous** physical activities in your leisure time?

_____ **hours per day**
_____ **minutes per day**

24. Again, think about only those physical activities that you did for at least 10 minutes at a time. During the **last 7 days**, on how many days did you do **moderate** physical activities like bicycling at a regular pace, swimming at a regular pace, and doubles tennis **in your leisure time**?

_____ **days per week**

☐ No moderate activity in leisure time → ***Skip to PART 5: TIME SPENT SITTING***

25. How much time did you usually spend on one of those days doing **moderate** physical activities in your leisure time?
_____ **hours per day**
_____ **minutes per day**

PART 5: TIME SPENT SITTING

The last questions are about the time you spend sitting while at work, at home, while doing course work and during leisure time. This may include time spent sitting at a desk, visiting friends, reading or sitting or lying down to watch television. Do not include any time spent sitting in a motor vehicle that you have already told me about.

26. During the **last 7 days**, how much time did you usually spend **sitting** on a **weekday**?

_____ **hours per day**
_____ **minutes per day**

27. During the **last 7 days**, how much time did you usually spend **sitting** on a **weekend day**?

_____ **hours per day**
_____ **minutes per day**

This is the end of the questionnaire, thank you for participating.

LONG LAST 7 DAYS SELF-ADMINISTERED version of the IPAQ. Revised October 2002.

REFERENCES

[1] Liberato MV, Nascimento AS, Ayers SD, et al. (2012) Medium chain fatty acids are selective peroxisome proliferator activated receptor (PPAR) gamma activators and pan-PPAR partial agonists. *PLoS One* 7:e36297.
[2] Turner N, Hariharan K, TidAng J, et al. Enhancement of Muscle Mitochondrial Oxidative Capacity and Alterations in Insulin Action Are Lipid Species Dependent Potent Tissue-Specific Effects of Medium-Chain Fatty Acids. *Diabetes* 2009;58(11):2547-54.
[3] Petersen K, Dufour S, Befroy D, et al. Impaired Mitochondrial Activity in the Insulin-Resistant Offspring of Patients with Type 2 Diabetes. New Engl J Medicine 2004;350(7):664-71.
[4] Wein S, Wolffram S, Schrezenmeir J, et al. Medium-chain fatty acids ameliorate insulin resistance caused by high-fat diets in rats. *Diabetes Metab Res Rev* 2009;25(2):185-94.
[5] Han J, Deng B, Sun J, et al. Effects of dietary medium-chain triglyceride on weight loss and insulin sensitivity in a group of moderately overweight free-living type 2 diabetic Chinese subjects. *Metabolism* 2007;56(7):985-91
[6] Yost TJ, Eckel RH. Hypocaloric feeding in obese women: metabolic effects of medium-chain triglyceride substitution. *Am J Clin Nutr* 1989;49: 326-30.
[7] Eckel R, Hanson A, Chen A, et al. Dietary substitution of medium-chain triglycerides improves insulin-mediated glucose metabolism in NIDDM subjects. *Diabetes* 1992;41(5):641-7.
[8] Yost T, Erskine J, Gregg T, et al. Dietary substitution of medium chain triglycerides in subjects with non-insulin-dependent diabetes mellitus in an ambulatory setting: impact on glycemic control and insulin-mediated glucose metabolism. *J Am Coll Nutr* 1994;13(6):615–22.
[9] St-Onge MP, Bosarge A, Goree L. Medium chain triglyceride oil consumption as part of a weight loss diet does not lead to an adverse metabolic profile when compared to olive oil. *J Am Coll Nutr* 2008;27(5):547-52.
[10] Airhart S, Cade T, Jiang H, et al. A Diet Rich in Medium-chain Fatty Acids Improves Systolic Function and Alters the Lipidomic Profile in Patients with Type 2 Diabetes: a Pilot Study. *J Clin Endocrinol Metab* 2016;101(2):504-12.
[11] Seaton T, Welle S, Warenko M, Campbell R. Thermic effect of medium-chain and long-chain triglycerides in man. *Am J Clin Nutr* 1986;44(5):630-4.
[12] St-Onge MP, Ross R, Parsons WD, Jones PJ. Medium-chain triglycerides increase energy expenditure and decrease adiposity in overweight men. *Obes Res* 2003;11(3):395-402.
[13] St-Onge MP, Jones PJ. Greater rise in fat oxidation with medium-chain triglyceride consumption relative to long-chain triglyceride is associated with lower initial body weight and greater loss of subcutaneous adipose tissue. *International Journal of Obesity* 2003;27(12):1565-71.
[14] Kasai M, Nosaka N, Maki H, et al. Effect of dietary medium- and long-chain triacylglycerols (MLCT) on accumulation of body fat in healthy humans. *Asia Pac J Clin Nutr* 2003;12(2):151-60.
[15] Siener R, Ehrhardt C, Bitterlich N, Metzner C. Effect of a fat spread enriched with medium-chain triacylglycerols and a special fatty acid-micronutrient combination on cardiometabolic risk factors in overweight patients with diabetes. *Nutr Metabol* 2011;8(1):21.
[16] St-Onge MP, Mayrsohn B, O'Keeffe M, et al. Impact of medium and long chain triglycerides consumption on appetite and food intake in overweight men. *Eur J Clin Nutr* 2014;68(10):1134-40.
[17] Goran MI. Ethnic-specific pathways to obesity-related disease: the Hispanic vs. African-American paradox. *Obesity* (Silver Spring) 2008;16(12):2561-5.
[18] Bunt J, Krakoff J, Ortega E, et al. Acute insulin response is an independent predictor of type 2 diabetes mellitus in individuals with both normal fasting and 2-h plasma glucose concentrations. *Diabetes Metab Res Rev* 2007;23:304-10.

[19] Jensen CC, Cnop M, Hull RL, et al. Beta-cell function is a major contributor to oral glucose tolerance in high-risk relatives of four ethnic groups in the U.S. Diabetes 2002;51(7):2170-8.
[20] Velasquez-Mieyer PA, Cowan PA, Umpierrez GE, et al. Racial differences in glucagon-like peptide-1 (GLP-1) concentrations and insulin dynamics during oral glucose tolerance test in obese subjects. *Int J Obes Relat Metab Disord* 2003;27(11):1359-64.
[21] NCD Risk Factor Collaboration. Trends in adult body-mass index in 200 countries from 1975 to 2014: a pooled analysis of 1698 population-based measurement studies with 19·2 million participants. *Lancet* 2016;387:1377
[22] Ferrannini E, Mari A. Beta cell function and its relation to insulin action in humans: a critical appraisal. *Diabetologia* 2004;47: 943-56.
[23] Polidori DC, Bergman RN, Chung ST, Sumner AE. Hepatic and Extra-Hepatic Insulin Clearance Are Differentially Regulated: Results from a Novel Model-Based Analysis of Intravenous Glucose Tolerance Data. *Diabetes* 2016 March 18 [ePub ahead of print].
